# An interpretable multimodal morphomolecular model refines progression-risk stratification in early-stage endometrioid endometrial carcinoma

**DOI:** 10.3389/fcell.2026.1945984

**Published:** 2026-10-19

**Authors:** Jinxin Li, Qiong Tang, Jinyang Chen, Qiaoqiao Chen, Bochen Liu, Qingran Kong, Jingzhang Ji, Qi Zhao

**Affiliations:** 1 Ningbo Institute of Innovation for Combined Medicine and Engineering, The Affiliated Lihuili Hospital of Ningbo University, Ningbo, China; 2 Section of Quality Management, Science and Education, Jiaocheng District Center for Disease Control and Prevention, Ningde, China; 3 School of Electronic Science and Technology, Xiamen University, Xiamen, Fujian, China; 4 Department of Obstetrics and Gynecology, Nanjing Drum Tower Hospital, Affiliated Hospital of Medical School, Nanjing University, Nanjing, China; 5 College of Life Sciences, Heilongjiang University, Harbin, Heilongjiang, China; 6 West China Second University Hospital, Sichuan University, Chengdu, China; 7 School of Laboratory Medicine and Life Sciences, Wenzhou Medical University, Wenzhou, China

**Keywords:** computational pathology, endometrioid endometrial carcinoma, explainable artificial intelligence, multimodal artificial intelligence, progression-free survival, risk stratification, transcriptomics, whole-slide imaging

## Abstract

**Background:**

Risk stratification remains incomplete for patients with FIGO stage I–II endometrioid endometrial carcinoma (EEC) despite clinicopathological assessment and molecular subtyping. Interpretable integration of diagnostic tissue morphology with molecular programmes may provide complementary information for progression-risk assessment.

**Methods:**

We developed a prototype-resolved interpretable survival model (PRISM), a multimodal framework integrating haematoxylin and eosin-stained whole-slide images with RNA-seq-derived pathway programmes for progression-free survival risk assessment. PRISM was developed in 287 eligible TCGA-UCEC patients with EEC, comprising a training cohort (n = 229) and a held-out internal validation cohort (n = 58). After model specification was fixed, PRISM was retrained on all 287 cases and evaluated without further tuning in an independent CPTAC-UCEC cohort of 63 patients with EEC. Prototype–pathway alignment provided tissue- and pathway-level interpretation of model predictions.

**Results:**

Among 350 patients with EEC and matched multimodal data, PRISM achieved C-indices of 0.770, 0.751 and 0.745 in the TCGA training, internal validation and CPTAC external validation cohorts, respectively. In CPTAC-UCEC, the 36-month time-dependent AUC was 0.727, compared with 0.685 for the clinicopathological and molecular subtype baseline model. In a complementary fixed-horizon analysis, adding PRISM to the clinicopathological model increased the 36-month AUC in CPTAC-UCEC from 0.609 to 0.719. The PRISM risk score remained associated with progression-free survival after multivariable adjustment. Prototype-resolved analyses linked higher-risk predictions to localised histomorphological patterns and pathway programmes related to proliferation, cell-cycle activity, glycolysis, hypoxia, focal adhesion and epithelial–mesenchymal transition.

**Conclusion:**

PRISM showed prognostic discrimination in an exploratory independent external evaluation and provided a biologically interpretable framework for integrating histopathology and transcriptomics to refine progression-risk assessment in early-stage EEC. Further validation in larger, prospective, multi-institutional cohorts is warranted to establish its generalisability and clinical utility.

## Introduction

Endometrial carcinoma (EC) is the most common gynaecological malignancy among women in high-income countries, and its incidence continues to rise (How et al., 2024; Corr et al., 2025). Approximately 80% of patients are diagnosed with International Federation of Gynecology and Obstetrics (FIGO) stage I–II disease (Akada et al., 2021; Makabe et al., 2024). Although most patients with localised EC are cured by surgery, 10%–20% develop distant recurrence, which is typically incurable (Volinsky-Fremond et al., 2024). Identifying this high-risk subset before recurrence remains difficult.

Endometrioid endometrial carcinoma (EEC) is the predominant histological subtype of EC, accounting for approximately 85% of cases (Sznurkowski et al., 2023). Current risk assessment in early-stage EEC relies on clinicopathological variables, including FIGO stage, histological grade, myometrial invasion and lymphovascular space invasion (LVSI), together with molecular subtype information (Concin et al., 2025). Molecular classification has substantially improved EC risk assignment, but clinically relevant uncertainty remains (Cancer Genome Atlas Research et al., 2013; Stelloo et al., 2016; Horeweg et al., 2023). Histological grading can be subject to interobserver variability (Gemer et al., 2009), while LVSI assessment requires distinction from morphological mimics (Peters et al., 2019). Molecular subtype information also requires interpretation alongside tumour morphology, spatial heterogeneity and clinical features (Gatius et al., 2018; Leon-Castillo, 2023). Routine haematoxylin and eosin (H&E)-stained whole-slide images (WSIs) contain rich information on tumour architecture, invasive growth, stromal reaction and immune infiltration, but these spatial phenotypes are difficult to quantify reproducibly by visual review alone (Fremond et al., 2023; Volinsky-Fremond et al., 2024).

Digital pathology has enabled molecular and prognostic modelling from diagnostic H&E-stained tissue, including molecular-class and recurrence-risk prediction in EC and prognostic modelling across cancer types (Fremond et al., 2023; Volinsky-Fremond et al., 2024; Wang et al., 2024). Multimodal pathology–omics approaches connect tissue morphology with genomic or transcriptomic programmes (Chen et al., 2022a; Chen et al., 2022b; Lipkova et al., 2022). Recent virtual spatial profiling frameworks have also shown that H&E images encode spatial molecular and cellular information associated with prognosis and treatment response (Li et al., 2026; Shulman et al., 2026). Pathway-aware models such as SurvPath and PAMT have enabled fine-grained modelling of associations between local histological regions and molecular programmes (Jaume et al., 2024; Yan et al., 2026). Patch-level modelling preserves local information, but estimating large numbers of patch–pathway interactions can be difficult in disease-specific cohorts with limited matched multimodal data. Prototype-based multimodal models have begun to use compact histological and pathway representations for survival prediction (Raza et al., 2025). Prototype learning groups recurrent local patterns into a small set of shared tissue states, reducing the number of patch-level representations passed to multimodal fusion while retaining heterogeneity at the level of recurrent morphological patterns.

In this study, we developed the prototype-resolved interpretable survival model (PRISM), a multimodal framework integrating routine H&E-stained WSIs with transcriptome-derived pathway programmes for progression-free survival (PFS) risk assessment in patients with FIGO stage I–II EEC. PRISM was designed to capture prognostically informative tissue heterogeneity through a compact set of shared morphological prototypes and to align these prototypes with pathway-level molecular programmes. The model was developed and internally validated in TCGA-UCEC, then refitted on all eligible TCGA-UCEC cases and evaluated without further tuning in the independent CPTAC-UCEC cohort. We further assessed its incremental prognostic value beyond clinicopathological variables and molecular subtype and examined whether its prototype-resolved outputs linked model-defined risk to spatially localised histomorphology and progression-associated pathway programmes.

## Methods

### Study design, cohorts and data sources

This retrospective prognostic study included patients with FIGO stage I–II endometrioid endometrial carcinoma with available clinicopathological data, PFS information, RNA-seq profiles and diagnostic H&E-stained WSIs. TCGA-UCEC was used for model development and internal validation, and CPTAC-UCEC was used as an independent external validation cohort. Patients were excluded if they had recurrent or previously treated disease, advanced-stage disease, missing or inadequate survival information, histologies outside the prespecified eligibility criteria, unavailable RNA-seq data, or unavailable or inadequate WSIs. PFS was calculated from available clinical time-to-event fields as the interval from initial pathological diagnosis to the earliest documented event, including disease progression, recurrence, new tumour event or death from any cause. Observed follow-up duration was summarised as median and interquartile range. Median potential follow-up and its 95% confidence interval were estimated using the reverse Kaplan–Meier method. Patients who were alive and without a documented progression-related event were censored at the last available follow-up. Survival-related fields from TCGA-UCEC and CPTAC-UCEC were harmonised to this endpoint definition before analysis. Clinicopathological and molecular variables included age, harmonised FIGO stage, histological grade, molecular subtype, depth of myometrial invasion, residual tumour status, radiation therapy, and LVSI where available. All included patients had matched H&E-stained WSIs, RNA-seq profiles, clinicopathological variables, and PFS information. Baseline cohort characteristics are reported in Table 1. Detailed PFS event composition, follow-up summaries, and available LVSI information are provided in Supplementary Table S1, and cohort selection and dataset partitioning are shown in Supplementary Figure S1.

**TABLE 1:** Baseline characteristics of the study cohorts.

Characteristic	TCGA-UCEC N = 287	CPTAC-UCEC N = 63	Overall N = 350
Age, years	63.0 (56.0, 71.0)	62.0 (56.0, 66.0)	62.0 (56.0, 70.0)
FIGO stage			
Stage I	256 (89.2%)	56 (88.9%)	312 (89.1%)
Stage II	31 (10.8%)	7 (11.1%)	38 (10.9%)
Histological grade			
G1	83 (29%)	26 (41%)	109 (31%)
G2	92 (32%)	29 (46%)	121 (35%)
G3	112 (39%)	8 (13%)	120 (34%)
Molecular subtype			
CN-high	30 (10%)	8 (13%)	38 (11%)
CN-low	106 (37%)	32 (51%)	138 (39%)
MSI	101 (35%)	18 (29%)	119 (34%)
POLE	29 (10%)	4 (6.3%)	33 (9.4%)
Unknown	21 (7.3%)	1 (1.6%)	22 (6.3%)
Myometrial invasion			
<50%	165 (57%)	37 (59%)	202 (58%)
≥50%	99 (34%)	19 (30%)	118 (34%)
Unknown	23 (8.0%)	7 (11%)	30 (8.6%)
Radiation therapy			
No	162 (56%)	29 (46%)	191 (55%)
Yes	113 (39%)	34 (54%)	147 (42%)
Unknown	12 (4.2%)	0 (0%)	12 (3.4%)
Residual tumor			
R0	222 (77%)	47 (75%)	269 (77%)
R1	4 (1.4%)	0 (0%)	4 (1.1%)
RX	61 (21%)	16 (25%)	77 (22%)
PFS events, n (%)	53 (18.5%)	8 (12.7%)	61 (17.4%)
Median potential follow-up, months (95% CI)	35.8 (31.7–42.1)	47.8 (31.8–52.7)	36.9 (33.8–46.1)

Continuous variables are presented as median (interquartile range), except for median potential follow-up, which was estimated using the reverse Kaplan–Meier method and is reported with 95% confidence intervals; categorical variables are presented as n (%). Median observed follow-up was 36.0 months (IQR, 20.0–63.0) in TCGA-UCEC and 48.0 months (IQR, 24.0–58.0) in CPTAC-UCEC. Detailed PFS event composition is provided in Supplementary Table S1. Molecular subtypes were classified according to the TCGA, integrated molecular classification: CN-high, copy-number high; CN-low, copy-number low; MSI, microsatellite instability; POLE, DNA, polymerase epsilon exonuclease-domain mutant.

TCGA-UCEC patients were divided at the patient level into a TCGA training cohort and a TCGA internal validation cohort using a predefined stratified 8:2 split. Model development, hyperparameter selection and internal model selection used the TCGA-UCEC training and internal validation split. After the model specification, hyperparameters, preprocessing workflow, and checkpoint-selection rule had been fixed, the final PRISM model was trained on all multimodally complete patients in the TCGA full cohort and evaluated once in the CPTAC external validation cohort (Dou et al., 2020). CPTAC-UCEC was not used for model training, feature selection, hyperparameter tuning, checkpoint selection, model selection or outcome-guided threshold optimisation. The locked model configuration, training parameters, preprocessing settings, and checkpoint-selection criteria are summarised in Supplementary Table S11.

### Multimodal data preprocessing

RNA-seq profiles were processed into pathway-level transcriptomic inputs. Genes with low or sparse expression were filtered out, followed by log2 (x + 1) transformation and gene-wise standardisation. Hallmark gene sets and KEGG Legacy canonical pathways were obtained from MSigDB 2025.1. Hs (Subramanian et al., 2005; Kanehisa et al., 2016). Gene-level expression profiles were organised into 236 pathway-level tokens, yielding a 236 × 6292 pathway-token input matrix for each patient. Gene filtering rules, retained-gene sets, pathway mapping and standardisation parameters were estimated using TCGA-UCEC development data and then applied without refitting to held-out TCGA-UCEC and CPTAC-UCEC samples.

For the pathology modality, H&E-stained WSIs were matched to clinical and RNA-seq data at the patient level. Slides with severe blur, scanning artefacts or insufficient tissue were excluded. Tissue regions were identified by low-magnification masking, and 256 × 256-pixel patches were extracted at an equivalent 20× scale. Patch embeddings were generated using a pathology encoder adapted to endometrial carcinoma histopathology using TCGA-UCEC development data only; CPTAC-UCEC slides were not used during encoder adaptation (Chen et al., 2024). Patch embeddings from the single diagnostic slide were processed into a fixed-length representation comprising 500 WSI tokens, each with 384 dimensions. Additional details on gene-filtering thresholds, standardisation, slide quality control, encoder adaptation, and WSI tokenisation are provided in the Supplementary Material. The corresponding locked preprocessing and model parameters are summarised in Supplementary Table S11.

### PRISM architecture and model training

PRISM was designed as a prototype-guided multimodal survival model integrating WSI-derived morphological representations and pathway-level transcriptomic representations for patient-level PFS risk prediction. The pathology branch compressed WSI-derived token sequences through an adaptive prototype learning (APL) bottleneck into four learnable morphological prototypes. The transcriptomic branch mapped RNA-seq-derived pathway tokens into a compatible latent space. A pathway–prototype alignment module linked pathway-level transcriptomic programmes with prototype-level morphological representations. The resulting multimodal representation was passed to a Cox output head to generate a continuous PRISM risk score, with higher scores indicating a greater risk of a PFS event.

Model training optimised a joint objective comprising Cox partial-likelihood loss, cross-modal alignment loss, and APL regularisation terms. In the APL-ablated model, the prototype bottleneck and associated APL regularisation terms were removed. Models were trained using AdamW with cosine learning-rate scheduling. Harrell’s C-index in the TCGA internal validation cohort was used for checkpoint selection, while time-dependent AUCs were evaluated as complementary performance measures. After the architecture, hyperparameters, preprocessing workflow, training procedure, and checkpoint-selection rule had been fixed, the final PRISM model was retrained using the TCGA full cohort and evaluated in CPTAC-UCEC without further tuning. Detailed architecture, loss-function, optimisation, and checkpoint-selection settings are provided in the Supplementary Material and Supplementary Table S11.

To assess sensitivity to the original data partition, PRISM, the APL-ablated model, and the WSI-only model were retrained across five independently generated stratified 8:2 TCGA training/internal-validation splits using randomly selected split seeds. For each split, data-dependent preprocessing parameters were estimated using the corresponding TCGA training partition and applied unchanged to its internal validation partition. The same model configuration, optimisation procedure, and checkpoint-selection rule were used across all five splits. CPTAC-UCEC remained excluded from repeated-split training and model selection. Training and internal-validation C-indices across the five splits were summarised as mean ± SD (Supplementary Table S6).

### Model performance evaluation

Model performance was evaluated using Harrell’s C-index, time-dependent ROC AUC, calibration analysis, and decision-curve analysis. Time-dependent AUCs at 12, 36, and 60 months were estimated using inverse probability of censoring weighting (IPCW). The 36-month horizon was specified as the primary time point for cross-cohort comparisons, including external evaluation in CPTAC-UCEC (Heagerty et al., 2000). C-index and IPCW-adjusted 36-month AUC estimates were reported with 95% confidence intervals obtained from 5,000 patient-level bootstrap resamples.

Split-specific discrimination was evaluated in the TCGA training and internal validation cohorts. Performance after model refitting in the TCGA full cohort was interpreted as apparent performance, and external performance was evaluated in the CPTAC external validation cohort. Calibration curves assessed agreement between predicted and observed 36-month event probabilities. Quantitative calibration of PRISM in the TCGA full and CPTAC external validation cohorts was additionally assessed using the calibration slope and calibration-in-the-large, with 95% confidence intervals obtained from 5,000 patient-level bootstrap resamples. Decision-curve analysis was used to estimate net benefit across threshold probabilities at the same 36-month prediction horizon. The model specifications used for the incremental, calibration, and decision-curve analyses are described below.

### Comparator and incremental clinical analyses

For discrimination analyses, PRISM was compared with an APL-ablated model, a WSI-only model, a pathway-only model, a clinical–molecular comparator, a clinical–molecular + FIGO comparator, and a FIGO stage-only reference benchmark. The clinical–molecular comparator included age, histological grade, depth of myometrial invasion, and molecular subtype. The clinical–molecular + FIGO comparator additionally incorporated harmonised FIGO stage (Stage I versus Stage II). Both clinical comparator models were fitted using the same penalised Cox proportional-hazards framework. FIGO stage-only was evaluated using a univariable Cox model. LVSI was not incorporated because harmonised LVSI information was unavailable in TCGA-UCEC.

The incremental prognostic value of PRISM was evaluated using two complementary analyses. First, comparative discrimination within the time-to-event framework was assessed using paired differences in C-index and IPCW-adjusted 36-month time-dependent AUC. PRISM was compared with the clinical–molecular and clinical–molecular + FIGO comparators in the TCGA full cohort and CPTAC external validation cohort. Both models were evaluated within the same 5,000 patient-level bootstrap resamples. Point estimates were calculated in the original cohort as PRISM minus the corresponding comparator, and 95% confidence intervals were obtained from the percentile distribution of the paired bootstrap differences (Eertink et al., 2022).

Separately, an exploratory fixed-horizon analysis assessed the add-on value of PRISM using four logistic regression models at 36 months: a clinical model including age, histological grade, and depth of myometrial invasion; a clinical–molecular model additionally including molecular subtype; a clinical + PRISM model; and a clinical–molecular + PRISM model. All four models were fitted in the 36-month-evaluable subset of the TCGA full cohort and applied without refitting to the corresponding CPTAC subset. The binary endpoint was defined as the occurrence of a PFS event within 36 months: patients who experienced an event within this period were classified as having an event, whereas those followed for at least 36 months without an event were classified as event-free. Patients censored before 36 months without a documented PFS event were excluded because their event status at the prespecified horizon could not be ascertained, consistent with previous fixed-horizon prognostic modelling studies (Eertink et al., 2022). This formulation enabled direct assessment of the discriminatory gain associated with adding PRISM at a clinically interpretable time point; however, because it does not incorporate partial follow-up from patients censored before 36 months, it was considered complementary to the primary IPCW-adjusted time-to-event analysis. Add-on value was quantified as the change in fixed-horizon AUC after adding PRISM to the clinical and clinical–molecular models. At the same horizon, calibration curves assessed agreement between predicted and observed event probabilities for the clinical–molecular + PRISM model, while decision-curve analysis compared its net benefit with that of the clinical–molecular comparator and the treat-all and treat-none strategies (Vickers and Elkin, 2006).

A separate clinical–molecular + PRISM Cox model was used to construct a nomogram for estimating PFS probabilities at 36 and 60 months. This model included age, histological grade, depth of myometrial invasion, molecular subtype, and the continuous PRISM risk score. For the feature-attribution analysis, predictor-specific contributions were calculated on the Cox linear-predictor scale for each patient and summarised as the mean absolute contribution across patients in the TCGA internal validation and CPTAC external validation cohorts. For categorical predictors represented by multiple model terms, term-specific contributions were aggregated at the original predictor level before summarisation.

### Risk stratification and prognostic association

Risk stratification was evaluated using Kaplan–Meier curves and log-rank tests. The primary PRISM risk-score cut-point was selected in the TCGA full cohort using maximally selected rank statistics with minprop = 0.10, requiring each resulting group to contain at least 10% of patients. The resulting cut-point of 2.5717 was applied unchanged to the CPTAC external validation cohort without outcome-based optimisation. Because the cut-point was selected and evaluated in the same TCGA full cohort, the corresponding TCGA log-rank P value was interpreted as descriptive. As a sensitivity analysis independent of outcome information at the cut-point selection step, the median PRISM risk score in the TCGA full cohort was used as the cut-point and applied unchanged to CPTAC. Risk groups were used only for Kaplan–Meier visualisation and log-rank comparison; C-index, time-dependent AUC, and Cox regression analyses were based on the continuous PRISM risk score.

Prognostic associations were assessed using univariable and multivariable Cox proportional hazards models. Given the limited number of PFS events in CPTAC-UCEC, the external multivariable analysis was considered exploratory. Multivariable models included age, histological grade, molecular subtype, depth of myometrial invasion, radiation therapy and residual tumour status. The continuous PRISM risk score was modelled using restricted cubic spline terms with 3 degrees of freedom (Austin, 2025). The proportional hazards assumption was assessed using Schoenfeld residuals (Hess, 1995).

### Prototype-resolved interpretation and sensitivity analyses

Prototype-resolved interpretation assessed whether PRISM risk predictions could be linked to pathway-level transcriptomic programmes and spatially recognisable histomorphology. From the final PRISM model, we extracted prototype assignment scores, activation maps, pathway-level representations and pathway-prototype association scores. Pathway-prototype associations were quantified using PRISM-generated softmax scores, standardised within each pathway for visualisation, and compared across cohorts as detailed in the Supplementary Material. Risk-associated pathway programmes were evaluated using PRISM-derived pathway-programme scores in both cohorts, and directional concordance of risk-group differences was assessed between TCGA-UCEC and CPTAC-UCEC. These model-derived scores were interpreted as pathway-level morphomolecular associations.

Representative prototype-associated patches were reviewed by a gynaecological pathologist to assign descriptive morphological interpretations to the learned prototypes. This review was used for interpretive labelling only and was not used for model training, risk-score generation, threshold selection or outcome prediction. Patches with high assignment to each prototype were examined across TCGA-UCEC and CPTAC-UCEC cases. All four learned prototypes were retained in the predictive model. P1–P3 were emphasised in the primary interpretation analyses because they showed recurrent histomorphological patterns and pathway associations, whereas P4 was reviewed separately because it predominantly captured low-content or artefact-associated regions. Spatial activation maps were generated by projecting prototype scores back to WSI coordinates. Patient-level prototype burden was defined as the mean prototype assignment among the top 10 representative patches for each patient-prototype pair. Sensitivity analyses across K = 4, K = 8 and K = 16 prototypes assessed pathway-prototype allocation, pathway-anchor concentration, prototype-burden differences and held-out discrimination.

Within-prototype cross-cohort concordance of pathway–prototype alignment scores was assessed separately for P1–P4 using Pearson correlation across the complete evaluated pathway set. Spearman correlation was used to assess cross-cohort directional concordance of risk-group differences in pathway-programme scores. Differences in pathway scores and prototype burden between risk groups were assessed using two-sided Wilcoxon rank-sum tests. Prototype-burden P values were reported as nominal P values, and Benjamini–Hochberg correction was applied separately within each cohort across P1–P3. P4 was excluded from this correction because it was not retained as a primary biological interpretation anchor. False discovery rate correction was applied to multiple pathway-programme comparisons as specified in the Supplementary Material.

### Statistical analysis and reporting guidelines

Statistical tests were two-sided unless otherwise specified. Multiple-testing correction was applied only to the prespecified families of comparisons described above and in the Supplementary Material. Analyses were performed using R version 4.3.3 and Python version 3.10; detailed package versions are provided in Supplementary Table S12. This retrospective prognostic modelling study was reported in accordance with the STROBE statement for cohort observational studies (von Elm et al., 2007) and the REMARK recommendations for tumour marker prognostic studies (McShane et al., 2005).

## Results

### Cohort construction and study overview

A total of 350 patients with FIGO stage I–II endometrioid endometrial carcinoma met the prespecified eligibility criteria, including 287 patients from TCGA-UCEC and 63 patients from CPTAC-UCEC. The TCGA full cohort was divided into a training cohort of 229 patients and an internal validation cohort of 58 patients. Cohort selection and dataset partitioning are shown in Supplementary Figure S1. Baseline clinicopathological characteristics are summarised in Table 1. The TCGA-UCEC and CPTAC-UCEC cohorts had similar median ages and broadly comparable distributions of myometrial invasion and residual tumour status, but differed in histological grade, molecular subtype composition, and use of radiation therapy. TCGA-UCEC included 53 PFS events among 287 patients (18.5%), whereas CPTAC-UCEC included 8 PFS events among 63 patients (12.7%). Median potential follow-up estimated using the reverse Kaplan–Meier method was 35.8 months (95% CI, 31.7–42.1) in TCGA-UCEC and 47.8 months (95% CI, 31.8–52.7) in CPTAC-UCEC. Median observed follow-up was 36.0 months (IQR, 20.0–63.0) and 48.0 months (IQR, 24.0–58.0), respectively. Detailed PFS event composition, follow-up summaries, and available LVSI information are reported in Supplementary Table S1. The study workflow and the PRISM architecture for integrating WSI-derived morphological representations with RNA-seq-derived pathway programmes are summarised in Figure 1.

**FIGURE 1:**
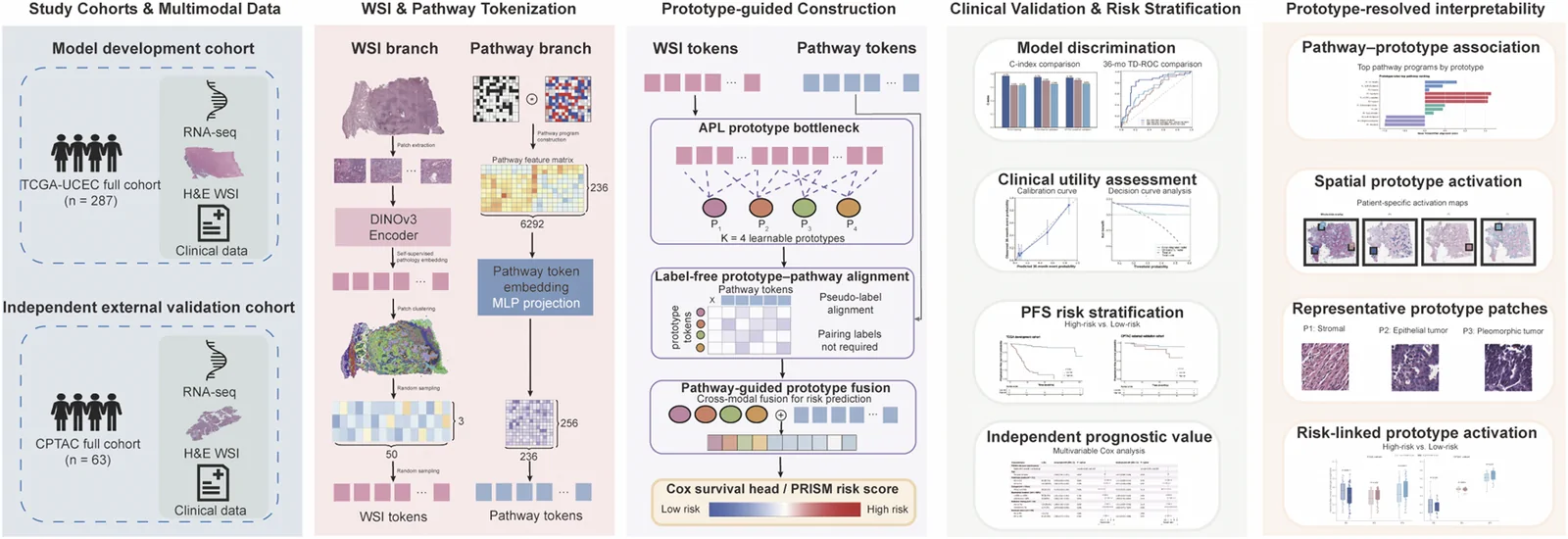
Study design and overview of the prototype-resolved PRISM framework. The schematic summarises the development, validation, and interpretation workflow of PRISM for PFS risk stratification in FIGO stage I–II EEC. The TCGA-UCEC cohort was used for model development and internal validation, whereas the CPTAC-UCEC cohort was retained for independent external validation. Matched H&E-stained WSIs and RNA-seq profiles were processed into WSI-derived morphological representations and pathway-level transcriptomic programmes. PRISM generated patient-level PFS risk scores through prototype-resolved integration of paired histopathological and transcriptomic information. Model performance was evaluated using discrimination metrics, calibration and decision-curve analyses, PFS risk stratification, and independent prognostic analysis. Prototype-resolved interpretation was used to visualise pathway-prototype associations, spatial activation patterns, and representative histomorphological regions.

### PRISM showed PFS risk discrimination in internal validation and exploratory external evaluation

The comparison of full and lightweight encoder fine-tuning strategies is shown in Supplementary Figure S2. The selected full fine-tuning strategy was used in the subsequent PRISM analyses. PRISM achieved C-indices of 0.770, 0.751, and 0.745 in the TCGA training, TCGA internal validation, and CPTAC external validation cohorts, respectively (Figure 2A). After refitting in the TCGA full cohort, the apparent C-index was 0.818 (Supplementary Table S2). In the TCGA internal validation cohort, the C-index was 0.751 (95% CI, 0.597–0.869) for PRISM and 0.649 (95% CI, 0.456–0.837) for the clinical–molecular comparator. In CPTAC-UCEC, the corresponding estimates were 0.745 (95% CI, 0.649–0.880) and 0.655 (95% CI, 0.459–0.805), respectively (Supplementary Table S3).

**FIGURE 2:**
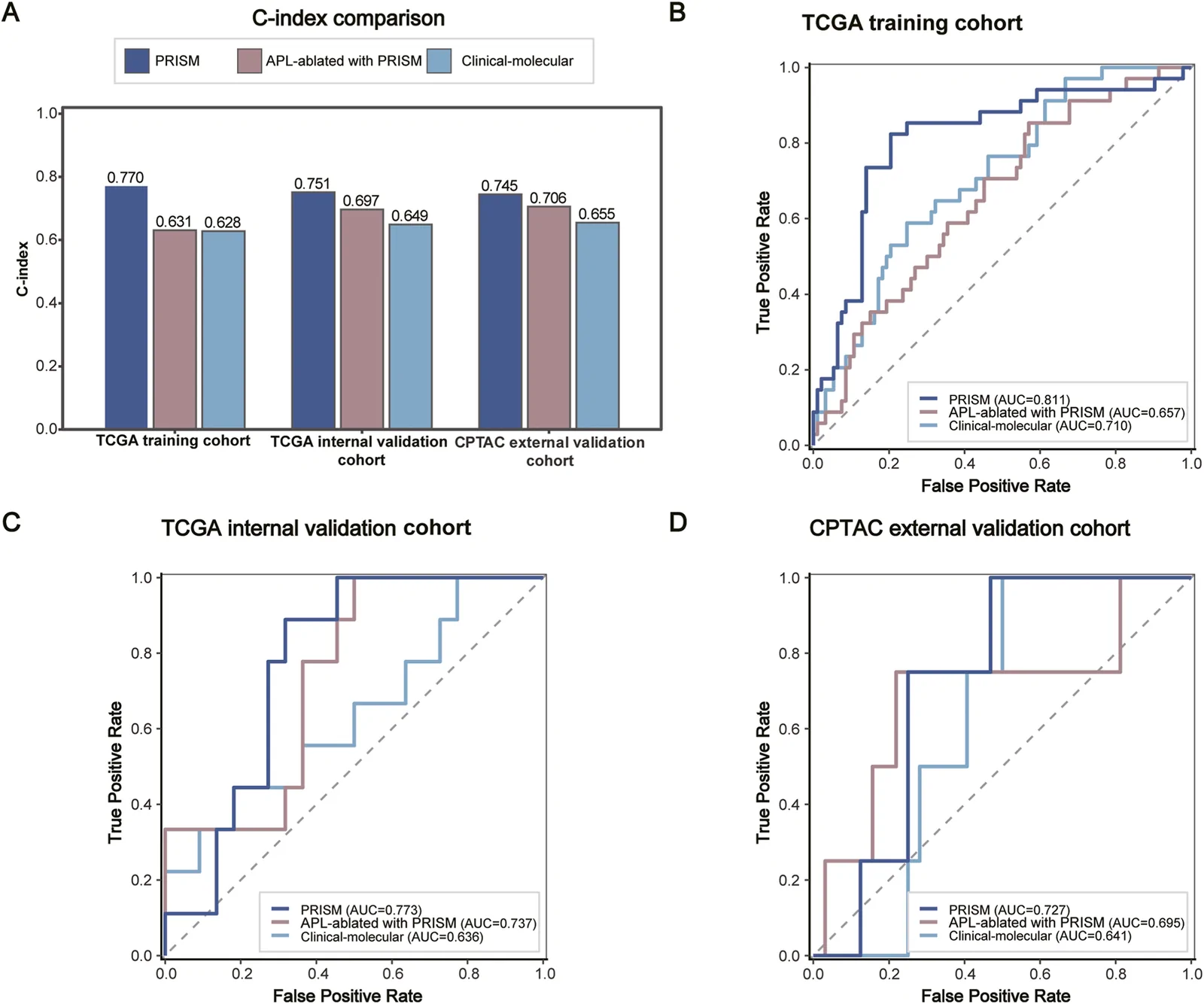
Model discrimination across the TCGA training, TCGA internal validation, and CPTAC external validation cohorts. **(A)** Harrell’s C-index estimates for PRISM, the APL-ablated model, and the clinical–molecular comparator in the TCGA training cohort, TCGA internal validation cohort, and CPTAC external validation cohort. The clinical–molecular comparator included age, histological grade, depth of myometrial invasion, and molecular subtype. **(B–D)** IPCW-adjusted 36-month time-dependent receiver operating characteristic (ROC) curves for the three models in the TCGA training cohort **(B)**, TCGA internal validation cohort **(C)**, and CPTAC external validation cohort. **(D)** Area under the curve (AUC) values are reported in the corresponding panel legends. The dashed diagonal line denotes the no-discrimination reference.

At 36 months, the IPCW-adjusted time-dependent AUCs for PRISM were 0.811, 0.773, and 0.727 in the TCGA training, TCGA internal validation, and CPTAC external validation cohorts, respectively (Figures 2B–D). The apparent AUC after refitting in the TCGA full cohort was 0.862. The corresponding values for the clinical–molecular comparator were 0.716, 0.640, 0.703, and 0.685 in the TCGA training, TCGA internal validation, TCGA full, and CPTAC external validation cohorts, respectively (Supplementary Table S4). In CPTAC-UCEC, the 95% confidence intervals for PRISM and the clinical–molecular comparator were 0.561–0.892 and 0.512–0.850, respectively, indicating uncertainty in the external estimates. The WSI-only model yielded high C-index estimates in the TCGA training and full cohorts (0.906 and 0.912, respectively), but its C-index decreased to 0.584 in CPTAC-UCEC. The pathway-only model achieved C-indices of 0.847, 0.727, 0.835, and 0.662 in the TCGA training, TCGA internal validation, TCGA full, and CPTAC external validation cohorts, respectively. The corresponding external C-index for the APL-ablated model was 0.706. Extended comparisons across model variants, cohorts, and time points are provided in Supplementary Figure S3 and Supplementary Tables S2–S4. Adding FIGO stage to the clinical–molecular comparator did not improve discrimination consistently. The C-index increased from 0.628 to 0.683 in the TCGA training cohort and from 0.639 to 0.664 in the TCGA full cohort, but decreased from 0.649 to 0.605 in the TCGA internal validation cohort and from 0.655 to 0.628 in CPTAC-UCEC. FIGO stage alone yielded C-indices of 0.519, 0.407, 0.504, and 0.488 in the four cohorts, respectively (Supplementary Table S2).

Across five independent TCGA splits, the mean internal-validation C-index was 0.7428 ± 0.0369 for PRISM, 0.6263 ± 0.0777 for the APL-ablated model, and 0.6563 ± 0.1012 for the WSI-only model (Supplementary Table S6), showing that the relative performance pattern was not restricted to the original split.

### Incremental clinical analyses of PRISM relative to clinical comparators

Paired bootstrap analysis was used to quantify comparative discrimination within the time-to-event framework. In the TCGA full cohort, PRISM showed a ΔC-index of 0.179 (95% CI, 0.067–0.288) and a Δ36-month IPCW-adjusted AUC of 0.159 (95% CI, 0.023–0.278) relative to the clinical–molecular comparator. Relative to the clinical–molecular + FIGO comparator, the corresponding differences were 0.154 (95% CI, 0.054–0.251) and 0.129 (95% CI, 0.002–0.245). In CPTAC-UCEC, the ΔC-index estimates were 0.090 (95% CI, −0.135–0.335) relative to the clinical–molecular comparator and 0.117 (95% CI, −0.079–0.340) relative to the clinical–molecular + FIGO comparator. The corresponding Δ36-month AUC estimates were 0.042 (95% CI, −0.244–0.294) and 0.116 (95% CI, −0.151–0.342) (Supplementary Table S5).

A complementary fixed-horizon analysis assessed the add-on value of PRISM among patients with ascertainable 36-month PFS status. In the 36-month evaluable subset of the TCGA full cohort (n = 158; 43 events within 36 months), the AUC was 0.607 for the clinical model, 0.697 for the clinical–molecular model, 0.896 for the clinical + PRISM model, and 0.905 for the clinical–molecular + PRISM model (Figure 3A). Adding PRISM increased the AUC by 0.289 relative to the clinical model and by 0.208 relative to the clinical–molecular model. In the corresponding 36-month evaluable subset of the CPTAC external validation cohort (n = 38; 6 events within 36 months), the AUCs were 0.609, 0.667, 0.719, and 0.755, respectively (Figure 3B). The AUC increases after adding PRISM were 0.110 relative to the clinical model and 0.088 relative to the clinical–molecular model (Figure 3C). Patients censored before 36 months without a documented PFS event were excluded because their 36-month event status could not be determined. These estimates therefore apply to the 36-month evaluable subsets and are not directly interchangeable with the IPCW-adjusted time-dependent AUC estimates based on the complete time-to-event data.

**FIGURE 3:**
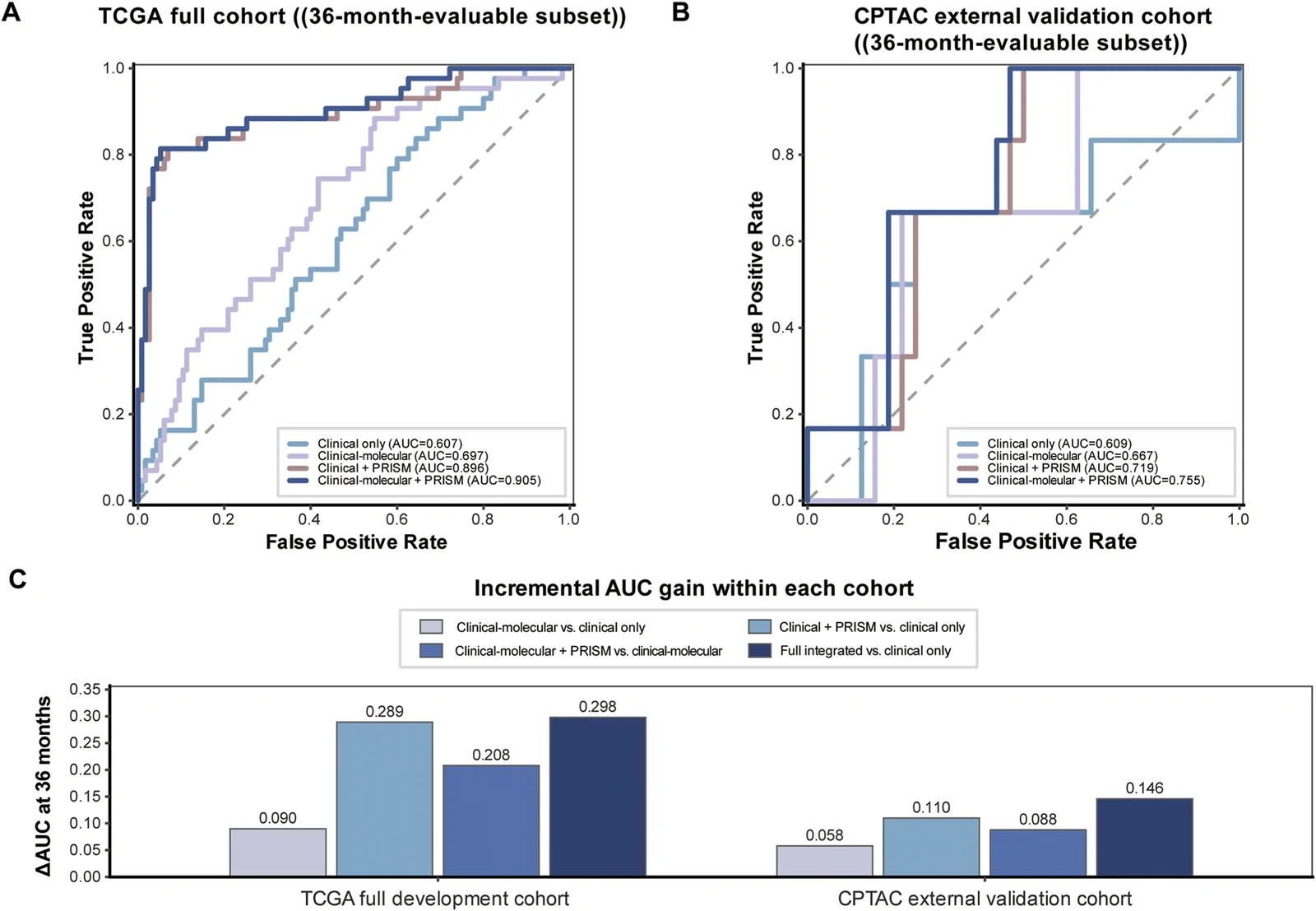
Incremental discrimination provided by PRISM in the exploratory 36-month fixed-horizon analysis. **(A)** Fixed-horizon receiver operating characteristic (ROC) curves for the clinical, clinical–molecular, clinical + PRISM, and clinical–molecular + PRISM models in the 36-month-evaluable subset of the TCGA full cohort (n = 158; 43 PFS events within 36 months). **(B)** Fixed-horizon ROC curves for the same models in the corresponding CPTAC external validation subset (n = 38; 6 PFS events within 36 months). The models were fitted in the TCGA subset and applied to CPTAC-UCEC without refitting. **(C)** Changes in 36-month fixed-horizon area under the curve (AUC) within each cohort. Bars represent the AUC differences between the clinical–molecular and clinical models, the clinical + PRISM and clinical models, the clinical–molecular + PRISM and clinical–molecular models, and the clinical–molecular + PRISM and clinical models. The clinical model included age, histological grade, and depth of myometrial invasion; the clinical–molecular model additionally included molecular subtype. Analyses were restricted to patients with ascertainable 36-month PFS status. AUC values are reported in the corresponding panel legends, and the dashed diagonal line denotes the no-discrimination reference.

Quantitative calibration assessment of PRISM showed an apparent 36-month calibration slope of 1.0000 (95% CI, 0.7895–1.2105) and calibration-in-the-large of −0.178 (95% CI, −0.455 to 0.055) in the TCGA full cohort. In CPTAC-UCEC, the calibration slope was 0.5631 (95% CI, 0.1266–0.9996), and calibration-in-the-large was −0.758 (95% CI, −1.931 to −0.004), indicating attenuation of the calibration slope and overall overprediction of absolute risk (Supplementary Table S10).

For the incremental clinical analysis, Supplementary Figure S4 presents 36-month calibration curves for the clinical–molecular + PRISM model and decision-curve analyses directly comparing its net benefit with that of the clinical–molecular comparator. The combined model showed higher net benefit over parts of the evaluated threshold-probability range in the TCGA cohorts. The CPTAC-UCEC decision curves were less stable, and no definitive conclusion regarding external clinical utility can be drawn from this cohort.

A clinical–molecular + PRISM Cox model was also used to construct a nomogram for estimating PFS probabilities at 36 and 60 months (Supplementary Figure S5A). On the Cox linear-predictor scale, the PRISM risk score had the largest mean absolute contribution among the included predictors in both the TCGA internal validation and CPTAC external validation cohorts (Supplementary Figures S5B,C).

### PRISM stratified PFS risk and remains associated with PFS after adjustment

Using the TCGA-derived cut-point of 2.5717, PRISM separated the TCGA full cohort into groups with different PFS distributions (log-rank P < 0.001; Figure 4A). This P value was interpreted descriptively because the cut-point was selected and evaluated in the same cohort. When the cut-point was applied unchanged to CPTAC-UCEC, the risk groups also showed different PFS distributions (log-rank P = 0.031; Figure 4B). In the median-based sensitivity analysis, PFS separation remained evident in the TCGA full cohort (log-rank P < 0.001). In CPTAC-UCEC, the log-rank P value was 0.107; all eight PFS events occurred in the median-defined high-risk group, whereas no events occurred in the low-risk group (Supplementary Figure S9).

**FIGURE 4:**
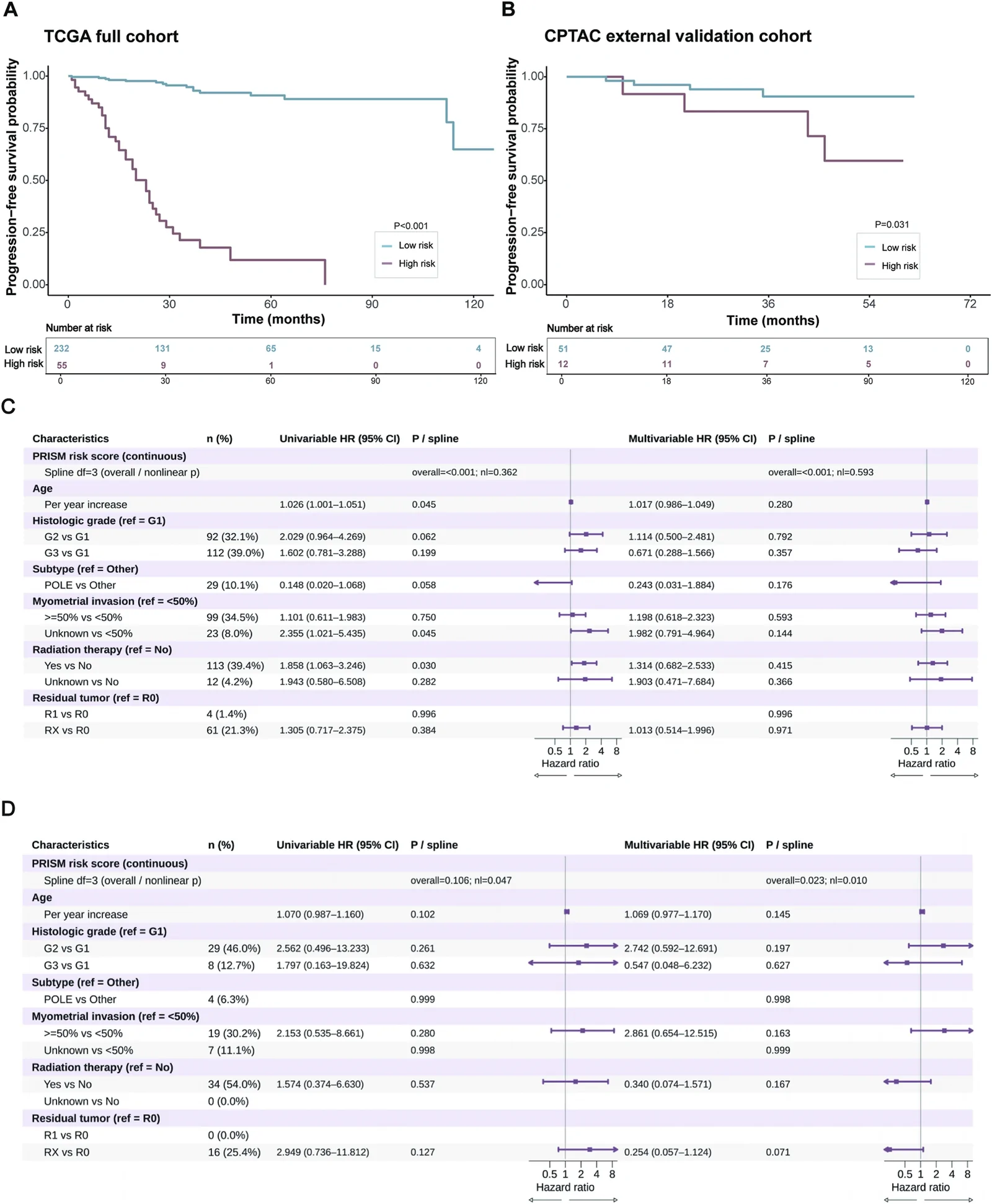
PRISM-based PFS risk stratification and prognostic associations. **(A,B)** Kaplan–Meier curves for PFS in the TCGA full cohort **(A)** and CPTAC external validation cohort. **(B)** Patients were classified into low- and high-risk groups using the PRISM risk-score cut-point of 2.5717, which was selected in the TCGA full cohort using maximally selected rank statistics with minprop = 0.10 and applied unchanged to CPTAC-UCEC. The TCGA log-rank *P* value is descriptive because cut-point selection and evaluation were performed in the same cohort. Log-rank tests were used to compare PFS distributions. **(C,D)** Univariable and multivariable Cox regression analyses in the TCGA full cohort **(C)** and CPTAC external validation cohort. **(D)** Cox analyses used the continuous PRISM risk score independently of the cut-point used for Kaplan–Meier visualisation. The PRISM risk score was modelled using restricted cubic spline terms with 3 degrees of freedom. Multivariable models adjusted for age, histological grade, molecular subtype, depth of myometrial invasion, radiation therapy, and residual tumour status. Forest plots show hazard ratios (HRs) and 95% confidence intervals (CIs) for covariates modelled with linear effects; overall and nonlinearity *P* values are reported for the spline-based PRISM risk score. The CPTAC-UCEC Cox analyses were considered exploratory because of the limited number of PFS events.

Because the continuous PRISM risk score was modelled using restricted cubic splines, its association with PFS was assessed using overall and nonlinearity tests instead of a single linear hazard ratio. In the TCGA full cohort, the PRISM risk score was associated with PFS in both univariable and adjusted models (overall P < 0.001 for both; Figure 4C). In CPTAC-UCEC, the risk score remained associated with PFS after adjustment (overall P = 0.023; Figure 4D). Given the small number of PFS events in CPTAC-UCEC, the external multivariable result was interpreted as exploratory.

Schoenfeld residual tests provided no statistical evidence of non-proportional hazards for the PRISM risk score in the TCGA full cohort (ρ = −0.102, χ^2^ = 0.536, P = 0.467) or CPTAC-UCEC (ρ = −0.135, χ^2^ = 0.112, P = 0.749; Supplementary Table S10).

### Prototype-resolved analyses reveal interpretable morphomolecular risk patterns

Pathway–prototype association analysis revealed structured relationships between morphological prototypes and pathway-level programmes in both cohorts (Figure 5A). The pooled cross-cohort correlation of pathway–prototype alignment scores was high (Pearson r = 0.91, P < 0.001; Figure 5B), although this pooled estimate was partly influenced by differences between prototypes. Within-prototype cross-cohort correlations were 0.730, 0.512, 0.728, and 0.444 for P1–P4, respectively (all P < 0.001), showing varying degrees of concordance at the individual-prototype level (Supplementary Table S7). In TCGA-UCEC, PRISM-defined high-risk tumours showed higher pathway-programme scores related to proliferation, cell-cycle activity, glycolysis, hypoxia, focal adhesion, and epithelial–mesenchymal transition. Low-risk tumours showed higher scores for metabolism- and homeostasis-associated programmes. The corresponding differences in CPTAC-UCEC were generally in the same direction, and the cross-cohort directional concordance analysis showed a positive association between pathway-score differences (Supplementary Figure S6).

**FIGURE 5:**
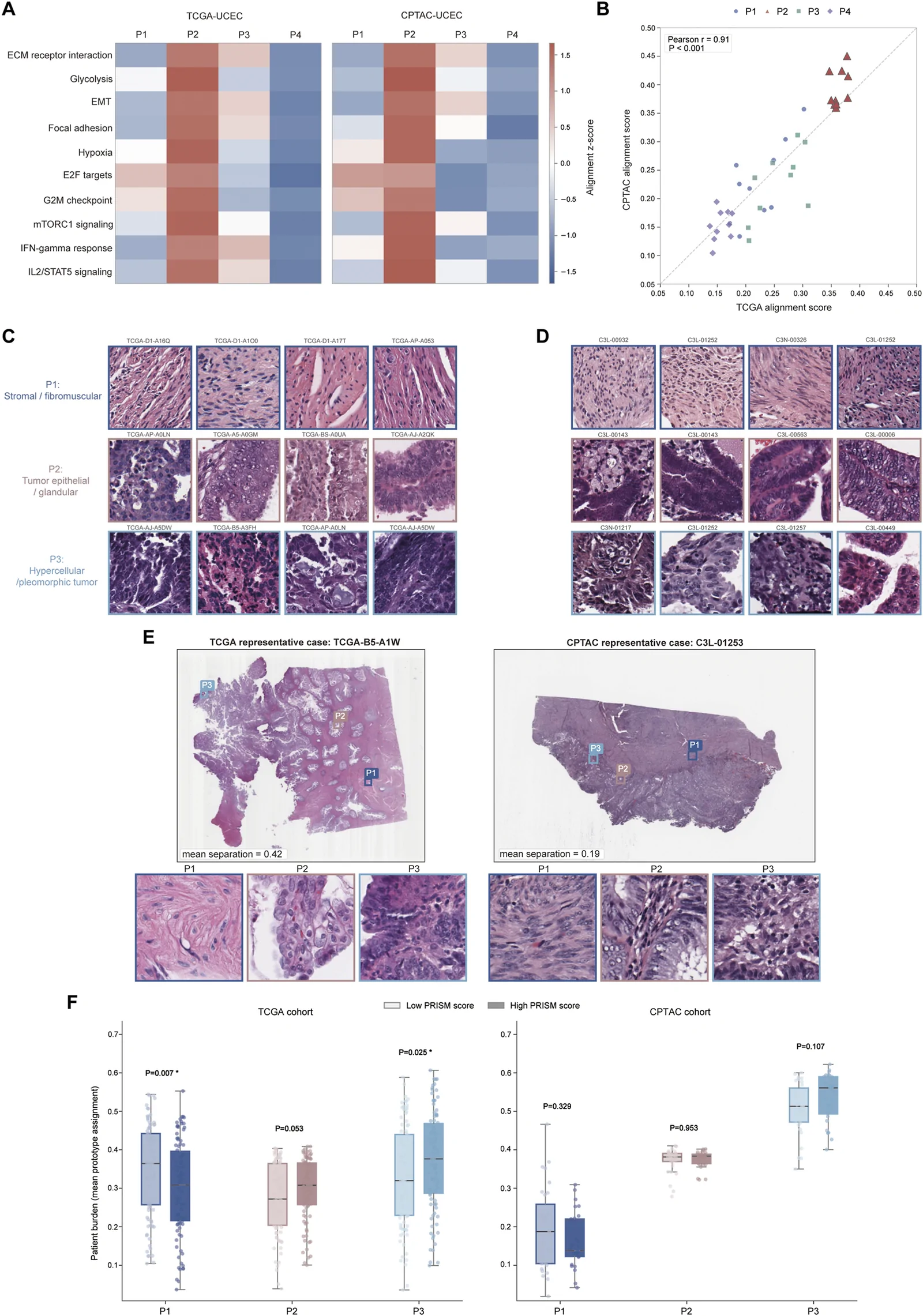
Prototype-resolved morphomolecular interpretation of PRISM-defined risk. **(A)** Heatmaps of pathway-prototype alignment scores in the TCGA-UCEC and CPTAC-UCEC cohorts. Rows indicate selected pathway programmes and columns indicate learned morphological prototypes. Colours represent row-wise standardised alignment z-scores. **(B)** Cross-cohort concordance of pathway-prototype alignment scores between TCGA-UCEC and CPTAC-UCEC. Each point represents a pathway-prototype pair and is coloured by prototype; the dashed diagonal line indicates equal alignment scores between cohorts. **(C)** Pathologist-reviewed representative TCGA-UCEC image patches assigned to the retained interpretable prototypes, corresponding to stromal/fibromuscular morphology, tumour epithelial/glandular morphology and hypercellular/pleomorphic tumour morphology. **(D)** Pathologist-reviewed representative CPTAC-UCEC image patches assigned to the same prototype categories. **(E)** Spatial localisation of prototype activation in representative TCGA-UCEC and CPTAC-UCEC whole-slide images, with enlarged examples of prototype-associated regions. **(F)** Patient-level prototype burden according to PRISM-defined risk group in the TCGA-UCEC and CPTAC-UCEC cohorts. Prototype burden was summarised from representative patient-prototype assignments as described in the Methods. P values were calculated using the group comparison test described in the Methods. P4 was included in alignment analyses but was treated as a low-content/artefact-associated prototype and was not used as a primary biological interpretation anchor. Prototype-burden analyses were used to interpret PRISM-defined risk groups and were not intended to establish a standalone morphology-only risk classifier. Pathologist review was used for descriptive prototype annotation only and was not used to train, tune or evaluate the prognostic model.

Pathologist review identified recurrent histomorphological patterns among the prototype-associated patches. P1 was enriched for stromal or fibromuscular morphology, P2 for tumour epithelial or glandular morphology, and P3 for hypercellular or pleomorphic tumour morphology in both cohorts (Figures 5C,D; Supplementary Table S8). P4 predominantly captured low-content, tissue-edge, or artefact-associated regions and was not retained as a primary biological interpretation anchor (Supplementary Figure S7D).

Spatial activation maps showed that P1–P3 occupied distinct regions within individual WSIs (Figure 5E). Cross-cohort pathway anchors and additional WSI overlays are shown in Supplementary Figures S5A–C. In the TCGA full cohort, high-risk tumours had lower P1 burden (nominal P = 0.007; BH-adjusted q = 0.021) and higher P3 burden (nominal P = 0.025; q = 0.038) than low-risk tumours. P2 burden was directionally higher in the high-risk group but was not statistically significant (nominal P = 0.053; BH-adjusted q = 0.053) (Figure 5F; Supplementary Table S9). The corresponding directions in CPTAC-UCEC were higher P1 burden in the low-risk group and higher P2 and P3 burden in the high-risk group. None of these comparisons remained significant after correction (q = 0.494, 0.953, and 0.321 for P1–P3, respectively). The CPTAC-UCEC burden analysis therefore provides directional external evaluation but not statistical replication.

Sensitivity analyses across K = 4, K = 8, and K = 16 showed that K = 4 provided a compact prototype–pathway structure while maintaining held-out discrimination comparable to that of the larger prototype sets (Supplementary Figure S8). These analyses linked PRISM risk predictions to pathway-associated morphological prototypes and spatially localised histological patterns, while the limited external cohort size constrained statistical replication of the prototype-burden associations.

## Discussion

FIGO stage I–II endometrioid endometrial carcinoma (EEC) presents an important opportunity for more precise risk stratification: most patients have favourable outcomes, while a clinically meaningful subset experiences progression, reflecting prognostic heterogeneity within this early-stage population (Cancer Genome Atlas Research et al., 2013; Kommoss et al., 2018; Horeweg et al., 2023; Concin et al., 2025). Resolving this heterogeneity beyond contemporary clinicopathological and molecular frameworks could support more individualised risk assessment. We developed PRISM as a proof-of-concept framework to test whether integrating H&E-derived morphology with transcriptome-derived pathway representations could capture residual prognostic variation beyond established risk factors. PRISM demonstrated prognostic discrimination in held-out TCGA internal validation and yielded favourable discrimination estimates in the independent CPTAC-UCEC cohort, providing initial evidence of cross-cohort transportability. Across five alternative TCGA splits, PRISM achieved a higher mean internal-validation C-index and lower between-split variability than the APL-ablated and WSI-only models, supporting its relative stability across data partitions. Complementary analyses highlighted the potential incremental prognostic information contributed by PRISM beyond the evaluated clinical–molecular comparator, while prototype-resolved analyses connected model-defined risk with cross-cohort-concordant pathway associations and recognisable histomorphology. Collectively, these findings support the feasibility of prototype-guided morphomolecular risk modelling in early-stage EEC and provide a foundation for broader validation and further evaluation of its clinical utility.

The clinical contribution of PRISM was examined through two analyses addressing distinct questions. The primary time-to-event analysis used Harrell’s C-index to assess overall risk ranking and IPCW-adjusted 36-month AUC to assess horizon-specific discrimination while accounting for censoring. Paired comparisons in the TCGA full cohort favoured PRISM over the clinical–molecular and clinical–molecular + FIGO comparators, and the corresponding CPTAC-UCEC estimates showed the same direction. A separate exploratory fixed-horizon analysis examined whether PRISM improved discrimination between patients who did and did not experience a PFS event within 36 months among those with ascertainable 36-month event status (Eertink et al., 2022). Under this binary formulation, adding PRISM increased AUC beyond both the clinical and clinical–molecular models in TCGA-UCEC and CPTAC-UCEC. This analysis provided a direct assessment of add-on discrimination at a clinically interpretable time point alongside the primary time-to-event evaluation. These incremental findings apply to the evaluated clinical–molecular comparator comprising age, histological grade, depth of myometrial invasion, and molecular subtype. Calibration and decision-curve analyses extended the 36-month evaluation to absolute-risk agreement and potential net benefit, while the external calibration results identified recalibration as an important step for transporting predicted risks across populations. The integrated nomogram and feature-attribution analysis further illustrated how the PRISM risk score contributed information within a combined clinicopathological and molecular risk model.

PRISM uses a compact, learnable morphological bottleneck to integrate histopathology with pathway-level transcriptomic representations. Earlier histology–genomic survival models integrated pathological and molecular information but commonly summarised histological features at the slide or patient level (Chen et al., 2022a; Chen et al., 2022b). Pathway-aware models, including SurvPath and PAMT, extended this approach by modelling relationships between pathway representations and local histological regions (Jaume et al., 2024; Yan et al., 2026). Directly modelling relationships between hundreds of WSI-derived tokens and hundreds of pathway representations produces a large interaction space that may be difficult to estimate in disease-specific cohorts with limited matched multimodal samples. In PRISM, each WSI-token sequence was first distilled into four adaptive morphological prototypes, after which cross-modal alignment was performed at the prototype–pathway level. These prototypes formed a shared set of morphological states across patients, while patch-to-prototype assignments retained links to local tissue regions and enabled spatial projection onto the WSI. The APL-ablated model retained direct pathway–patch modelling but showed lower discrimination and greater between-split variability, supporting the value of the prototype-learning formulation. This formulation provides a compact and interpretable approach to pathology–transcriptomics integration while retaining the regional traceability of model-derived risk.

The learned prototypes placed PRISM-derived risk within a recognisable morphological and molecular context. Pathologist review characterised P1 as predominantly stromal or fibromuscular morphology, P2 as tumour epithelial or glandular morphology, and P3 as hypercellular or pleomorphic tumour morphology. P4 predominantly represented low-content, tissue-edge, or artefact-associated regions and was treated separately from the principal biological interpretation anchors, while all four prototypes remained components of the predictive model. Spatial activation maps showed that P1–P3 occupied distinct regions within individual WSIs, linking patient-level predictions to regional tissue organisation. At the pathway level, high-risk tumours showed higher PRISM-derived scores for proliferation, cell-cycle progression, glycolysis, hypoxia, focal adhesion, and epithelial–mesenchymal transition, whereas low-risk tumours showed relatively higher scores for metabolism- and homeostasis-associated programmes. These pathway themes are consistent with previous studies of cell-cycle activity, metabolic reprogramming, hypoxia, focal adhesion, and epithelial–mesenchymal transition in endometrial cancer (Chiu et al., 2019; Liu et al., 2020; Salinas-Vera et al., 2022; Huang et al., 2023; Yan et al., 2024), as well as multi-omic and single-cell evidence of tumour-cell heterogeneity and microenvironmental crosstalk (Yu et al., 2022; Mao et al., 2024; Ren et al., 2024). The high pooled cross-cohort correlation of pathway–prototype alignment scores supported preservation of the broader morphomolecular organisation, and the positive within-prototype correlations provided additional evidence of concordance at the individual-prototype level. At the patient level, high-risk tumours in TCGA-UCEC showed lower P1 burden and higher P3 burden, consistent with a shift from stromal or fibromuscular representation towards hypercellular or pleomorphic tumour morphology; the corresponding CPTAC-UCEC comparisons showed a similar directional pattern. The concordance between pathway scores, prototype burden, and spatial localisation suggests that PRISM risk reflects coordinated differences in tissue morphology and pathway-level transcriptional programmes. These relationships provide specific targets for further investigation using spatial, single-cell, and functional approaches.

Several limitations should be considered. First, this retrospective study was based on selected public cohorts. Requiring matched WSIs, transcriptomic profiles, clinical variables, and PFS information may have affected cohort representativeness. PFS was reconstructed by harmonising available clinical time-to-event fields, and future studies using prospectively adjudicated endpoints will strengthen endpoint consistency. Second, CPTAC-UCEC included 63 patients and eight PFS events. The resulting external estimates for discrimination, calibration, decision curves, adjusted Cox associations, and prototype burden had limited precision, and the paired confidence intervals for incremental discrimination encompassed zero. Although the PRISM risk score remained associated with PFS after multivariable adjustment, the limited number of events relative to model complexity creates potential for overfitting and unstable adjusted estimates. Accordingly, the external multivariable analysis should be regarded as exploratory and should not be considered definitive evidence of independent prognostic value. Its findings require confirmation in larger independent cohorts with more outcome events. Third, performance after refitting in the TCGA full cohort represented apparent performance, and the outcome-guided risk cut-point was selected and evaluated in the same cohort. The corresponding TCGA risk-group analysis was therefore descriptive. The primary IPCW-adjusted analysis evaluated censoring-adjusted discrimination using the available time-to-event data, whereas the exploratory fixed-horizon analysis examined binary add-on discrimination among patients with ascertainable 36-month PFS status. The fixed-horizon estimates apply specifically to this evaluable subset. Fourth, the incremental-value conclusions apply to the evaluated clinical–molecular comparator, as harmonised LVSI information was unavailable in TCGA-UCEC. Each patient was represented by one diagnostic WSI, prototype-associated patches were reviewed by one gynaecological pathologist, and implementation of PRISM requires transcriptomic input. The pathway scores and prototype assignments also remain model-derived measures that warrant confirmation using complementary spatial, single-cell, and functional approaches. However, this study makes an important contribution by providing an externally evaluated proof of concept for prototype-guided integration of histopathology and transcriptomics in a clinically relevant early-stage population. The locked external evaluation, repeated-split analysis, complementary assessment of incremental value, and spatially traceable prototype outputs provide a well-defined starting point for larger-scale validation and clinical development.

The prototype–pathway representation raises a broader question: which morphomolecular relationships remain stable across cohorts, and which vary with tumour lineage or clinical context? Comparing prototype–pathway maps learned independently across institutions could reveal cross-cohort morphomolecular patterns that persist despite differences in patient populations, tissue processing, slide scanners, and sequencing platforms. Extending this analysis across cancer types could further determine whether recurrent adverse programmes, including proliferation, hypoxia, and epithelial–mesenchymal transition, are expressed through shared tissue states or through lineage-specific morphology. Spatial transcriptomic and single-cell data could identify the cellular populations and microenvironmental interactions underlying these conserved and context-dependent patterns. A comparative atlas of these relationships may improve model transportability and clarify how molecular risk is expressed in tissue architecture.

## Conclusion

In conclusion, PRISM provides an interpretable framework for refining progression-risk assessment in early-stage EEC through prototype-guided integration of histopathology and transcriptomics. By integrating diagnostic H&E-stained whole-slide histopathology with transcriptome-derived pathway programmes, PRISM linked residual progression risk to recognisable tissue patterns and biologically relevant pathway-programme signals. These findings support PRISM as an interpretable precision risk-refinement framework for multimodally profiled patients with early-stage EEC. Further validation in larger prospective and multi-institutional cohorts is needed before clinical deployment.

## Data Availability

No new raw sequencing, imaging or clinical data were generated in this study. TCGA-UCEC whole-slide images, transcriptomic profiles, clinicopathological annotations, molecular subtype information and survival-related clinical fields used to reconstruct progression-free survival are available through The Cancer Genome Atlas via the Genomic Data Commons Data Portal (https://portal.gdc.cancer.gov/projects/TCGA-UCEC). CPTAC-UCEC whole-slide images used for independent external validation were obtained from The Cancer Imaging Archive (TCIA) CPTAC-UCEC collection (https://www.cancerimagingarchive.net/collection/cptac-ucec/). CPTAC-UCEC transcriptomic and clinical data were downloaded using the Python cptac package (https://github.com/PayneLab/cptac). Additional clinical, follow-up and molecular subtype annotations were obtained or harmonised from CPTAC resources and associated supplementary materials where applicable. Pathway gene sets, including Hallmark gene sets and KEGG Legacy canonical pathways, were obtained from the Molecular Signatures Database (MSigDB 2025.1.Hs) via the GSEA website (https://www.gsea-msigdb.org/gsea/msigdb). Processed cohort lists, model-derived risk scores, prototype assignments, pathway-level features and analysis outputs are provided in the Supporting Information where applicable. The source code, preprocessing scripts, evaluation pipelines and model configuration files are publicly available at https://github.com/Jin918/PRISM-main.
