# Supplementary material for: An interpretable multimodal morphomolecular model refines progression-risk stratification in early-stage endometrioid endometrial carcinoma
Source: Front Cell Dev Biol. 2026 Oct 19;14:1945984. doi: 10.3389/fcell.2026.1945984 (PMC13724606; doi:10.3389/fcell.2026.1945984)
Supplement: Supplementary file 1 [file Supplementaryfile1.docx]

# **Supplementary Appendix**

## Supplementary Tables

# **Supplementary Table**

**Supplementary Table S1. Detailed clinicopathological and outcome characteristics of the study cohorts**

| **Characteristic** | **TCGA-UCEC N = 287** | **CPTAC-UCEC N = 63** | **Overall N = 350** |
| --- | --- | --- | --- |
| Age, years | 63.0 (56.0, 71.0) | 62.0 (56.0, 66.0) | 62.0 (56.0, 70.0) |
| Harmonised FIGO stage |  |  |  |
| Stage I | 256 (89.2%) | 56 (88.9%) | 312 (89.1%) |
| Stage II | 31 (10.8%) | 7 (11.1%) | 38 (10.9%) |
| Original cohort-reported FIGO stage |  |  |  |
| Stage I | 2 (0.7%) | 1 (1.6%) | 3 (0.9%) |
| Stage IA | 131 (45.6%) | 41 (65.1%) | 172 (49.1%) |
| Stage IB | 108 (37.6%) | 14 (22.2%) | 122 (34.9%) |
| Stage IC | 15 (5.2%) | 0 (0.0%) | 15 (4.3%) |
| Stage II | 22 (7.7%) | 7 (11.1%) | 29 (8.3%) |
| Stage IIA | 3 (1.0%) | 0 (0.0%) | 3 (0.9%) |
| Stage IIB | 6 (2.1%) | 0 (0.0%) | 6 (1.7%) |
| Histological grade |  |  |  |
| G1 | 83 (28.9%) | 26 (41.3%) | 109 (31.1%) |
| G2 | 92 (32.1%) | 29 (46.0%) | 121 (34.6%) |
| G3 | 112 (39.0%) | 8 (12.7%) | 120 (34.3%) |
| Molecular subtype |  |  |  |
| CN-high | 30 (10.5%) | 8 (12.7%) | 38 (10.9%) |
| CN-low | 106 (36.9%) | 32 (50.8%) | 138 (39.4%) |
| MSI | 101 (35.2%) | 18 (28.6%) | 119 (34.0%) |
| POLE | 29 (10.1%) | 4 (6.3%) | 33 (9.4%) |
| Unknown | 21 (7.3%) | 1 (1.6%) | 22 (6.3%) |
| Myometrial invasion |  |  |  |
| <50% | 165 (57.5%) | 37 (58.7%) | 202 (57.7%) |
| ≥50% | 99 (34.5%) | 19 (30.2%) | 118 (33.7%) |
| Unknown | 23 (8.0%) | 7 (11.1%) | 30 (8.6%) |
| Lymphovascular space invasion (LVSI) |  |  |  |
| Absent | — | 45 (71.4%) | — |
| Present | — | 17 (27.0%) | — |
| Unknown | — | 1 (1.6%) | — |
| Adjuvant radiation therapy |  |  |  |
| No | 162 (56.4%) | 29 (46.0%) | 191 (54.6%) |
| Yes | 113 (39.4%) | 34 (54.0%) | 147 (42.0%) |
| Unknown | 12 (4.2%) | 0 (0.0%) | 12 (3.4%) |
| Residual tumour |  |  |  |
| R0 | 222 (77.4%) | 47 (74.6%) | 269 (76.9%) |
| R1 | 4 (1.4%) | 0 (0.0%) | 4 (1.1%) |
| RX | 61 (21.3%) | 16 (25.4%) | 77 (22.0%) |
| Outcome characteristics |  |  |  |
| PFS events, n (%) | 53 (18.5%) | 8 (12.7%) | 61 (17.4%) |
| PFS event composition, n (% of PFS events) |  |  |  |
| Documented progression/new tumour event | 17 (32.1%) | 5 (62.5%) | 22 (36.1%) |
| Death without prior documented progression | 17 (32.1%) | 3 (37.5%) | 20 (32.8%) |
| PFS event, specific event type unavailable | 19 (35.8%) | 0 (0.0%) | 19 (31.1%) |
| Median potential follow-up, months (95% CI) | 35.8 (31.7-42.1) | 47.8 (31.8-52.7) | 36.9 (33.8-46.1) |

Continuous variables are presented as median (interquartile range), and categorical variables as n (%). Harmonised FIGO stage categories were used for the main analysis. LVSI information was unavailable in TCGA-UCEC and was therefore not included in cross-cohort comparator analyses. Median potential follow-up was estimated using the reverse Kaplan–Meier method and is reported with 95% confidence intervals. Separately, the median observed follow-up was 36.0 months (IQR, 20.0–63.0) in TCGA-UCEC and 48.0 months (IQR, 24.0–58.0) in CPTAC-UCEC.

**Supplementary Table S2. C-index comparison of PRISM, model variants, and clinical comparators**

| **Model** | **TCGA training cohort** | **TCGA internal validation cohort** | **TCGA full cohort** | **CPTAC external validation cohort** |
| --- | --- | --- | --- | --- |
| PRISM | 0.770 | 0.751 | 0.818 | 0.745 |
| APL-ablated | 0.631 | 0.697 | 0.656 | 0.706 |
| WSI-only | 0.906 | 0.737 | 0.912 | 0.584 |
| Pathway-only | 0.847 | 0.727 | 0.835 | 0.662 |
| Clinical–molecular | 0.628 | 0.649 | 0.639 | 0.655 |
| Clinical–molecular + FIGO | 0.683 | 0.605 | 0.664 | 0.628 |
| FIGO stage-only* | 0.519 | 0.407 | 0.504 | 0.488 |

The clinical–molecular comparator comprised age, histological grade, depth of myometrial invasion, and molecular subtype. The clinical–molecular + FIGO comparator additionally incorporated harmonised FIGO stage (Stage I versus Stage II) while maintaining the same modelling framework and evaluation procedure. FIGO stage-only was evaluated as a univariable Cox reference benchmark. Because the study population was restricted to FIGO stage I–II disease, the FIGO stage-only analysis should not be interpreted as an assessment of staging performance across the full FIGO spectrum. LVSI was not incorporated into cross-cohort comparator models because harmonised LVSI information was unavailable in TCGA-UCEC.

**Supplementary Table S3. C-index comparison across PRISM and clinical comparator models**

| **Model** | **TCGA training cohort** | **TCGA internal validation cohort** | **TCGA full cohort** | **CPTAC external validation cohort** |
| --- | --- | --- | --- | --- |
| PRISM | 0.770 (0.642–0.870) | 0.751 (0.597–0.869) | 0.818 (0.745–0.877) | 0.745 (0.649–0.880) |
| Clinical–molecular | 0.628 (0.536–0.716) | 0.649 (0.456–0.837) | 0.639 (0.559–0.718) | 0.655 (0.459–0.805) |
| Clinical–molecular + FIGO | 0.683 (0.604–0.760) | 0.605 (0.401–0.798) | 0.664 (0.593–0.735) | 0.628 (0.421–0.792) |
| FIGO stage-only * | 0.519 (0.471–0.579) | 0.407 (0.344–0.459) | 0.504 (0.455–0.544) | 0.488 (0.406–0.673) |

C-index estimates were calculated using survival concordance analysis and are presented with 95% confidence intervals derived from 5,000 patient-level bootstrap resamples. The clinical–molecular comparator comprised age, histological grade, depth of myometrial invasion, and molecular subtype. The clinical–molecular + FIGO comparator additionally incorporated harmonised FIGO stage (Stage I versus Stage II) using the same modelling framework. FIGO stage-only was evaluated as a univariable Cox model using harmonised FIGO stage as the sole predictor.

**Supplementary Table S4. 36-month time-dependent AUC comparison across PRISM and clinical comparator models**

| **Model** | **TCGA training cohort** | **TCGA internal validation cohort** | **TCGA full cohort** | **CPTAC external validation cohort** |
| --- | --- | --- | --- | --- |
| PRISM | 0.811 (0.696–0.881) | 0.773 (0.596–0.968) | 0.862 (0.775–0.935) | 0.727 (0.561–0.892) |
| Clinical–molecular | 0.716 (0.620–0.810) | 0.640 (0.406–0.845) | 0.703 (0.609–0.787) | 0.685 (0.512–0.850) |
| Clinical–molecular + FIGO | 0.728 (0.629–0.823) | 0.692 (0.466–0.884) | 0.733 (0.642–0.817) | 0.611 (0.366–0.859) |
| FIGO stage-only * | 0.487 (0.439–0.540) | 0.400 (0.318–0.474) | 0.469 (0.426–0.515) | 0.500 (0.371–0.687) |

The 36-month time-dependent AUCs were estimated using inverse probability of censoring weighting (IPCW), and 95% confidence intervals were obtained from 5,000 patient-level bootstrap resamples. The clinical–molecular comparator comprised age, histological grade, depth of myometrial invasion, and molecular subtype. The clinical–molecular + FIGO comparator additionally incorporated harmonised FIGO stage (Stage I versus Stage II) using the same modelling framework. FIGO stage-only was evaluated as a univariable Cox reference benchmark.

w

| **Comparison** | **Cohort** | **ΔC-index**  **(95% CI)** | **Δ36-month AUC**  **(95% CI)** |
| --- | --- | --- | --- |
| PRISM vs Clinical–molecular | TCGA full cohort | 0.179 (0.067–0.288) | 0.159 (0.023–0.278) |
| PRISM vs Clinical–molecular + FIGO | TCGA full cohort | 0.154 (0.054–0.251) | 0.129 (0.002–0.245) |
| PRISM vs Clinical–molecular | CPTAC external validation cohort | 0.090 (-0.135–0.335) | 0.042 (-0.244–0.294) |
| PRISM vs Clinical–molecular + FIGO | CPTAC external validation cohort | 0.117 (-0.079–0.340) | 0.116 (-0.151–0.342) |

**Supplementary Table S5. Paired bootstrap comparison of PRISM and clinical comparator models**

Δ values represent the observed performance differences in the original cohort, calculated as PRISM minus the corresponding clinical comparator. The 95% confidence intervals were derived from the percentile distribution of paired differences across 5,000 patient-level bootstrap resamples, with both models evaluated using identical resamples. Positive values favour PRISM.

**Supplementary Table S6. Repeated-split robustness analysis of PRISM and ablation models**

| **Model** | **TCGA training cohort C-index (mean ± SD)** | **TCGA internal validation cohort C-index (mean ± SD)** |
| --- | --- | --- |
| **PRISM** | **0.7783 ± 0.0488** | **0.7428 ± 0.0369** |
| APL-ablated | 0.6554 ± 0.0829 | 0.6263 ± 0.0777 |
| WSI-only | 0.9463 ± 0.0263 | 0.6563 ± 0.1012 |

C-index values are presented as mean ± SD across five independent random TCGA-UCEC splits (seeds: 58, 351, 479, 822, and 1198). CPTAC-UCEC was not used during repeated-split training, model selection, or checkpoint selection and was reserved exclusively for external validation.

**Supplementary Table S7. Within-prototype cross-cohort concordance of pathway–prototype alignment scores**

| **Prototype** | **Pearson r** | **95% CI** | **P value** |
| --- | --- | --- | --- |
| P1 | 0.730 | 0.664–0.784 | <0.001 |
| P2 | 0.512 | 0.412–0.601 | <0.001 |
| P3 | 0.728 | 0.662–0.783 | <0.001 |
| P4 | 0.444 | 0.336–0.541 | <0.001 |

Pearson correlations were calculated between TCGA-UCEC and CPTAC-UCEC pathway–prototype alignment scores for the same pathways separately within each prototype. P4 was included in the alignment analysis but was not retained as a primary biological interpretation anchor because it showed predominantly low-content morphological patterns.

**Supplementary Table S8. Prototype-resolved pathology-pathway interpretation of PRISM**

| **Prototype** | **Histopathological state** | **Representative pathway-linked programme** | **Risk-associated pattern** | **Integrated interpretation** |
| --- | --- | --- | --- | --- |
| P1 | Stromal  /fibromuscular morphology | E2F targets | Higher burden in low-risk tumours in both cohorts | Stromal/fibromuscular prototype showing a lower-risk-associated directional pattern across cohorts. |
| P2 | Epithelial  /glandular tumour morphology | Glycolysis | Higher burden in high-risk tumours in both cohorts, with a suggestive association in TCGA | Epithelial/glandular tumour prototype showing a higher-risk-associated pattern. |
| P3 | Hypercellular  /pleomorphic tumour morphology | ECM receptor interaction | Higher burden in high-risk tumours in both cohorts | Hypercellular/pleomorphic tumour prototype showing a higher-risk-associated directional pattern across cohorts. |

Risk-associated patterns indicate the observed direction of prototype-burden differences between PRISM-defined risk groups and should not be interpreted as evidence of statistical replication. Detailed statistical comparisons, including nominal P values and BH-adjusted q values, are provided in Supplementary Table S9. Representative pathway-linked programmes were selected from prototype-wise pathway association analyses and should be interpreted as pathway-associated programmes rather than single-pathway causal mechanisms. P4 was not retained as a primary biological interpretation anchor because it predominantly represented low-content, tissue-edge, and artefact-associated regions; extended P4 examples are shown in Supplementary Figure S7.

**Supplementary Table S9. Comparison of prototype burden between PRISM-defined risk groups**

| **Cohort** | **Prototype** | **Direction** | **P** | **BH-adjusted q** |
| --- | --- | --- | --- | --- |
| TCGA full cohort | P1 | Higher in low-risk | 0.007 | 0.021 |
| TCGA full cohort | P2 | Higher in high-risk | 0.053 | 0.053 |
| TCGA full cohort | P3 | Higher in high-risk | 0.025 | 0.038 |
| CPTAC external validation cohort | P1 | Higher in low-risk | 0.329 | 0.494 |
| CPTAC external validation cohort | P2 | Higher in high-risk | 0.953 | 0.953 |
| CPTAC external validation cohort | P3 | Higher in high-risk | 0.107 | 0.321 |

Prototype burden was defined for each patient and prototype as the mean prototype-assignment score of the 10 patches with the highest assignment scores. Between-group comparisons were performed using two-sided Mann–Whitney U tests. P values are nominal; q values were calculated using the Benjamini–Hochberg procedure across the three biologically interpreted prototypes (P1–P3) separately within each cohort. P4 was excluded from multiple-testing correction because it was not retained as a primary biological interpretation anchor.

**Supplementary Table S10. Calibration and proportional-hazards assessment of the PRISM model**

| **Metric** | **TCGA full cohort** | **CPTAC external validation cohort** |
| --- | --- | --- |
| Calibration slope (95% CI) | 1.0000 (0.7895–1.2105) | 0.5631 (0.1266–0.9996) |
| 36-month CITL (95% CI) | −0.178 (−0.455 to 0.055) | −0.758 (−1.931 to −0.004) |
| Schoenfeld ρ | −0.102 | −0.135 |
| Schoenfeld χ² | 0.536 | 0.112 |
| Schoenfeld *P* value | 0.467 | 0.749 |

Calibration slope and calibration-in-the-large (CITL) have ideal values of 1 and 0, respectively. Negative CITL values indicate overall overprediction. The 95% confidence intervals for calibration slope and CITL were estimated using 5,000 patient-level bootstrap resamples. Schoenfeld residual tests were used to assess the proportional-hazards assumption for the PRISM risk score.

| **Parameter** | **Locked value** | **Note** |
| --- | --- | --- |
| TCGA train/internal validation split | 8:2 | Patient-level split. |
| External validation cohort | CPTAC-UCEC | Not used for model development or selection. |
| Patch size | 256 × 256 pixels | Extracted at equivalent 20x scale. |
| WSI tokens per patient | 500 | Fixed-length pathology input. |
| WSI embedding dimension | 384 | Patch-level embedding dimension. |
| Pathway-token matrix | 236 × 6292 | 236 pathway tokens across 6,292 aligned genes. |
| Number of prototypes | 4 | Primary PRISM configuration. |
| Prototype temperature | 0.1 | Prototype-assignment temperature. |
| WSI branch depth | 2 | Locked model configuration. |
| Gene/pathway branch depth | 1 | Locked model configuration. |
| Dropout/stochastic-depth parameter | 0.3 | Locked regularization setting. |
| Optimizer | AdamW | Used for model training. |
| Initial learning rate | 0.0005 | Locked training value. |
| Cosine schedule lower-bound factor | 0.0003 | Locked training value. |
| Weight decay | 0.0001 | Locked training value. |
| Batch size | 128 | Locked training value. |
| TopK sampling parameter | 2 | Locked run configuration. |
| Contrastive supervision | Enabled | Contrastive loss flag = 1. |
| Checkpoint selection | Based on TCGA training/internal-validation performance | Selected using predefined TCGA development-stage criteria; CPTAC not used for model selection |
| Diversity regularization weight | 0.0 | Locked APL regularization value. |
| Balance regularization weight | 0.1 | Locked APL regularization value. |
| Random seed | 42 | Locked model seed. |
| View seed | 42 | Locked view-sampling seed. |
| Year-to-month conversion | 12.0 months per year | Used for survival-time conversion. |
| Risk-score cut-point | 2.5717 | Derived exclusively in the complete TCGA-UCEC cohort using maximally selected rank statistics (surv_cutpoint, minprop = 0.10). |
| Risk-group definition | >2.5717, high risk;  ≤2.5717, low risk | Applied unchanged to CPTAC-UCEC without outcome-based optimisation. |
| Prototype burden summary | Mean of top 10 prototype-map scores | Used for Fig. 5F representative-patch burden analysis. |

**Supplementary Table S11. Core locked model and preprocessing parameters.**

The final PRISM model used for CPTAC-UCEC external evaluation was trained on the complete TCGA-UCEC cohort after the model configuration had been fixed. CPTAC-UCEC was not used for model training, tuning, checkpoint selection, or threshold determination.

**Supplementary Table S12. Computational environment and main software packages.**

| **Software/package** | **Version or specification** |
| --- | --- |
| NVIDIA GPU | A100 80 GB |
| Python | 3.10 |
| torch | 2.9.0+cu128 |
| torchvision | 0.24.0+cu128 |
| torchaudio | 2.9.0+cu128 |
| numpy | 2.2.6 |
| pandas | 2.3.3 |
| scipy | 1.15.3 |
| matplotlib | 3.10.7 |
| scikit-learn | 1.7.2 |
| lifelines | 0.30.0 |
| OpenCV (cv2) | 4.12.0 |
| Pillow | 12.0.0 |
| einops | 0.8.1 |
| R | 4.3.3 |
| data.table | 1.17.8 |
| survival | 3.8.3 |
| ggplot2 | 3.5.2 |
| survminer | 0.5.1 |
| glmnet | 4.1.10 |
| timeROC | 0.4 |
| rms | 6.8.1 |
| grid | 4.3.3 |
