# Supplementary material for: An interpretable multimodal morphomolecular model refines progression-risk stratification in early-stage endometrioid endometrial carcinoma
Source: Front Cell Dev Biol. 2026 Oct 19;14:1945984. doi: 10.3389/fcell.2026.1945984 (PMC13724606; doi:10.3389/fcell.2026.1945984)
Supplement: Supplementary file 2 [file Supplementaryfile2.docx]

# **Supplementary Appendix**

## Supplementary Figures

# **Supplementary Figures**

**Supplementary Figure S1. Cohort selection and dataset partitioning.**
Flow diagram showing cohort selection and dataset partitioning for the TCGA-UCEC development cohort and the CPTAC-UCEC independent external validation cohort. Patients were screened using predefined criteria for treatment status, PFS availability, histology, FIGO stage, key clinicopathological annotations, RNA-seq availability, and WSI matching or quality control. Patient-level exclusions included recurrent or previously treated disease, missing or <30-day PFS time, serous or mixed histology, FIGO stage III/IV disease, missing age, tumour grade, or FIGO stage, and unavailable RNA-seq data. Additional exclusions during WSI matching and quality control included inadequate WSI quality or absence of a matched WSI. The final matched TCGA-UCEC cohort included 287 patients and was divided into a training set (n = 229) and an internal validation set (n = 58). The final matched CPTAC-UCEC cohort included 63 patients and was used as the independent external validation cohort.

**Supplementary Figure S2. Representative comparison of clustering maps derived from lightweight and fully fine-tuned pathology encoders.**
(A–C) Representative TCGA-UCEC case TCGA-EY-A2OM. (D–F) Representative TCGA-UCEC case TCGA-BS-A0UA. Panels (A) and (D) show the original haematoxylin and eosin-stained whole-slide image overviews. Panels (B) and (E) show clustering maps generated using features from the lightweight fine-tuned pathology encoder. Panels (C) and (F) show clustering maps generated using features from the fully fine-tuned pathology encoder. In these representative examples, features from the fully fine-tuned encoder showed more coherent spatial organisation and better preservation of broad histoanatomic structures than features from the lightweight fine-tuned encoder. Colours indicate unsupervised cluster assignments and do not represent ordinal intensity values.

**Supplementary Figure S3. Extended comparison of model discrimination across cohorts and prediction horizons.**

**(A) Harrell’s** C-index estimates for PRISM, the APL-ablated model, the WSI-only model, the pathway-only model, and the clinical–molecular comparator in the TCGA training cohort, TCGA internal validation cohort, TCGA full cohort, and CPTAC external validation cohort. Estimates in the TCGA full cohort represent apparent performance after model refitting. **(B) Heatmap** of IPCW-adjusted time-dependent AUC estimates for the same five models at 12, 36, and 60 months in the TCGA training, internal validation, and full cohorts, and at 36 months in the CPTAC external validation cohort. The clinical–molecular comparator included age, histological grade, depth of myometrial invasion, and molecular subtype. Numbers above the bars and within the heatmap cells are point estimates; higher values indicate better discrimination.

**Supplementary Figure S4. Calibration and decision-curve analyses for 36-month fixed-horizon PFS prediction.**

**(A) Calibration of the clinical–molecular + PRISM model in the TCGA full cohort. (B) Decision-curve comparison of the clinical–molecular + PRISM model with the clinical–molecular comparator in the TCGA full cohort. (C) Corresponding calibration in the TCGA internal validation cohort. (D) Corresponding decision-curve analysis in the TCGA internal validation cohort. (E) Corresponding calibration in the CPTAC external validation cohort. (F) Corresponding decision-curve analysis in the CPTAC external validation cohort.** The clinical–molecular comparator included age, histological grade, depth of myometrial invasion, and molecular subtype; the clinical–molecular + PRISM model additionally included the continuous PRISM risk score. Calibration plots show observed 36-month PFS event probabilities against predicted probabilities within groups of predicted risk. Vertical error bars indicate 95% confidence intervals, and the dashed diagonal line indicates ideal calibration. Decision-curve analyses show net benefit across threshold probabilities; the dashed and horizontal grey lines represent the treat-all and treat-none strategies, respectively. Analyses were restricted to patients whose event status at 36 months could be ascertained.

**Supplementary Figure S5. Nomogram and feature attribution of the fully integrated prognostic model.**
(A) Nomogram of the fully integrated prognostic model for estimating PFS probabilities at 36, 60 months. The model incorporated age, histological grade, myometrial invasion, molecular subtype, and the PRISM risk score. Each variable contributes points to the total score, which is mapped to the predicted PFS probability at each time point. (B) Feature attribution analysis of the fully integrated model in the TCGA internal validation set. (C) Feature attribution analysis of the fully integrated model in the CPTAC external validation cohort. Feature attribution was quantified as the mean absolute contribution of each variable to the model linear predictor.

**Supplementary Figure S6. Risk-associated pathway programmes and cross-cohort pathway-score concordance.**
(A) RNA-seq-derived pathway programmes with relatively higher activity in PRISM-defined high-risk tumours in the TCGA-UCEC cohort. Bars show standardised high-versus-low risk differences in bulk RNA-seq-derived pathway activity. (B) RNA-seq-derived pathway programmes with relatively higher activity in PRISM-defined low-risk tumours in the TCGA-UCEC cohort. Bars show standardised high-versus-low risk differences in bulk RNA-seq-derived pathway activity. (C) Directionally concordant pathway-score differences in the CPTAC-UCEC external validation cohort for representative high-risk- and low-risk-associated programmes identified in TCGA-UCEC. In CPTAC-UCEC, pathway scores were derived from PRISM pathway-prototype softmax outputs for selected prototype anchors rather than independently estimated bulk RNA-seq pathway activity. Positive values indicate higher scores in the high-risk group, whereas negative values indicate higher scores in the low-risk group. (D) Cross-cohort concordance between TCGA-UCEC RNA-seq-derived pathway activity differences and CPTAC-UCEC PRISM-derived pathway-score differences across evaluated pathway programmes. Each point represents one pathway programme; selected high-risk- and low-risk-associated programmes are labelled. CPTAC-UCEC, Clinical Proteomic Tumour Analysis Consortium uterine corpus endometrial carcinoma; PRISM, Prototype-Resolved Interpretable Survival Model; TCGA-UCEC, The Cancer Genome Atlas uterine corpus endometrial carcinoma.

**Supplementary Figure S7. Extended prototype-pathway anchors, spatial activation maps, and P4-associated patch review.**
(A) Mean cross-cohort alignment scores for selected prototype-associated pathway programmes. Bars indicate the average alignment score across the TCGA-UCEC and CPTAC-UCEC cohorts for representative pathways linked to each prototype. (B) Spatial activation maps for P1, P2, and P3 in a representative TCGA-UCEC case. The whole-slide overlay shows combined prototype activation, and separate maps show prototype-specific activation patterns. (C) Spatial activation maps for P1, P2, and P3 in a representative CPTAC-UCEC case. (D) Representative P4-associated patches from TCGA-UCEC and CPTAC-UCEC. P4-associated regions included fragmented peripheral or low-content tissue, peripheral edge-associated fragments, tissue-edge regions, and artefact/debris-associated patches, supporting its classification as a low-confidence background/artefact-associated prototype rather than a retained primary morphologic prototype.

**Supplementary Figure S8. Sensitivity analysis of prototype interpretability and discrimination across prototype numbers.**
(A) Prototype-pathway anchor allocation patterns across K = 4, K = 8, and K = 16. Heatmaps show standardised association z-scores between selected pathway programmes and learned prototypes. (B) Pathway-anchor concentration across K. Each point represents one pathway programme, and highlighted points indicate representative high-risk and low-risk anchors. Anchor concentration quantifies how strongly a pathway is allocated to a limited number of prototypes; the inset shows the near-uniform range for K = 8 and K = 16. (C) Relative prototype-burden sensitivity across K in the TCGA and CPTAC cohorts. Colours indicate within-K scaled high-minus-low differences in patient-level prototype burden between PRISM-defined risk groups. Asterisks indicate prototypes with statistically significant group differences. (D) Held-out discrimination across K in the TCGA internal validation and CPTAC external validation cohorts. Boxplots show the distribution of C-index values for each K setting. CPTAC, Clinical Proteomic Tumour Analysis Consortium; PRISM, Prototype-Resolved Interpretable Survival Model; TCGA, The Cancer Genome Atlas.

**Supplementary Figure S9. Median-based sensitivity analysis of PRISM-defined PFS risk stratification.**

**(A) Kaplan–Meier curve for progression-free survival (PFS) in the TCGA full cohort. (B) Corresponding Kaplan–Meier curve in the CPTAC external validation cohort.** As a sensitivity analysis independent of outcome information at the cut-point selection step, the median PRISM risk score in the TCGA full cohort was used to define low- and high-risk groups and was applied unchanged to CPTAC-UCEC. The TCGA analysis therefore used 144 low-risk and 143 high-risk patients, whereas the CPTAC analysis included 48 low-risk and 15 high-risk patients. Log-rank tests were used to compare PFS distributions, and the corresponding P values are shown in each panel. All eight PFS events in CPTAC-UCEC occurred in the median-defined high-risk group. Numbers at risk are displayed below the Kaplan–Meier curves.
