# Supplementary material for: An interpretable multimodal morphomolecular model refines progression-risk stratification in early-stage endometrioid endometrial carcinoma
Source: Front Cell Dev Biol. 2026 Oct 19;14:1945984. doi: 10.3389/fcell.2026.1945984 (PMC13724606; doi:10.3389/fcell.2026.1945984)
Supplement: Supplementary file 3 [file Supplementaryfile3.docx]

# Supplementary Appendix

## Supplementary Methods

# Supplementary Methods

## 1. Patient-level multimodal matching

Cohort selection, patient-level exclusion criteria, and dataset partitioning are summarized in Supplementary Fig. S1. All cohort counts were tabulated at the patient level. Clinical annotations, progression-free survival (PFS) information, RNA-seq profiles, and diagnostic hematoxylin and eosin-stained whole-slide images (WSIs) were harmonized using patient identifiers before dataset partitioning, ensuring that all slides and molecular data from the same patient were assigned to the same analytical subset.

When multiple diagnostic slides were available for one patient, slide-level features were processed before patient-level aggregation. The CPTAC-UCEC cohort was not used for model development, hyperparameter tuning, checkpoint selection, or risk-threshold selection for model development. The TCGA-UCEC and CPTAC-UCEC cohorts were interpreted in the context of established genomic and proteogenomic resources for endometrial carcinoma(Cancer Genome Atlas Research et al., 2013; Dou et al., 2020).

## 2. Pathway-token construction

The main Methods describe RNA-seq preprocessing and the generation of pathway-level transcriptomic inputs. Here, we provide the mathematical definition of the pathway-token representation. Pathway programs were constructed from MSigDB Hallmark gene sets and KEGG canonical pathways(Ogata et al., 1999; Subramanian et al., 2005; Liberzon et al., 2015). For model input construction, pathway information was not collapsed into one scalar value per pathway. Instead, each pathway was represented as a pathway-masked gene-expression vector.

Let $x_{i}\in\mathbb{R}^{G}$ denote the gene-expression vector for patient $i$, where $G=6292$, and let $M\in\{0,1{\}}^{P\times G}$ denote the pathway-gene membership matrix, where $P=236$. Gene-wise standardization was performed using training-set statistics:

$$z_{i,g}=\frac{x_{i,g}-\mu_{g}}{\sigma_{g}+\epsilon}, X_{i,p,g}^{\mathrm{path}}=M_{p,g}z_{i,g}.$$

(S1)

Here, $\mu_{g}$ and $\sigma_{g}$ denote the training-set mean and standard deviation for gene $g$, respectively, $M_{p,g}$ is the binary pathway membership indicator, and $X_{i}^{\mathrm{path}}\in\mathbb{R}^{236\times6292}$ is the patient-level pathway-by-gene token matrix. The same pathway definitions, gene order, and training-derived normalization parameters were used for external CPTAC-UCEC evaluation.

Scalar pathway activity values used for risk-associated pathway analyses were derived separately for visualization and group comparison. These scalar pathway summaries were not identical to the full pathway-token matrix used as the model input.

## 3. WSI token construction

Tissue regions were identified using low-magnification tissue masking, and patch extraction was restricted to tissue-containing regions. Patches were extracted at an equivalent 20× physical scale with a fixed size of 256 x 256 pixels.

Patch-level embeddings were generated using the pathology encoder used in the final feature-generation pipeline. Self-supervised domain adaptation was performed using TCGA-UCEC development data only; CPTAC-UCEC slides were not used during encoder adaptation. Full and lightweight fine-tuning strategies were compared using WSI feature-organization assessment, and the final downstream analyses used the fully fine-tuned embeddings. Each patient-level pathology input contained 500 WSI tokens, each represented by a 384-dimensional embedding.

## 4. APL bottleneck and training objective

The attention-based prototype learning (APL) bottleneck compressed WSI tokens into learnable morphologic prototype representations. Let $X_{i}\in\mathbb{R}^{N\times D}$ denote the WSI-token matrix for patient $i$, where $N=500$ and $D=384$. Let $P\in\mathbb{R}^{K\times D}$ denote the learnable prototype codebook. Assignment scores between WSI tokens and prototypes were computed as:

$$A_{i,n,k}=\frac{exp\{\left( X_{i,n}W_{q} \right)^{\top}\left( P_{k}W_{k} \right)/\tau\}}{\sum_{k'=1}^{K} \exp\{\left( X_{i,n}W_{q} \right)^{\top}\left( P_{k'}W_{k} \right)/\tau\}}.$$

(S2)

The prototype-level representation for prototype $k$ was obtained by assignment-weighted pooling:

$$Z_{i,k}^{\mathrm{proto}}=\frac{\sum_{n=1}^{N} A_{i,n,k}X_{i,n}}{\sum_{n=1}^{N} A_{i,n,k}+\epsilon}.$$

(S3)

The main optimization target was the Cox partial log-likelihood computed from the model risk score(Cox, 1972). For patient $i$, let $\theta_{i}$ denote the model risk score, $t_{i}$ the follow-up time, and $\delta_{i}\in\{0,1\}$ the event indicator. The Cox loss was:

$$\mathcal{L}_{\mathrm{cox}}=-\frac{1}{N}\sum_{i=1}^{N} \delta_{i}\left[ \theta_{i}-log\left( \sum_{j:t_{j}\geq t_{i}} \exp\left( \theta_{j} \right) \right) \right].$$

(S4)

The overall training objective was summarized as:

$$\mathcal{L}_{\mathrm{total}}=\mathcal{L}_{\mathrm{cox}}+I_{\mathrm{contrast}}\mathcal{L}_{\mathrm{contrast}}+\lambda_{\mathrm{div}}\mathcal{L}_{\mathrm{div}}+\lambda_{\mathrm{bal}}\mathcal{L}_{\mathrm{bal}}.$$

(S5)

Here, $\mathcal{L}_{\mathrm{contrast}}$ denotes the optional cross-modal contrastive loss, and $\mathcal{L}_{\mathrm{div}}$ and $\mathcal{L}_{\mathrm{bal}}$ denote the APL diversity and assignment-balance regularization terms, respectively. In APL-ablated models, APL-related regularization terms were disabled. Core locked model and preprocessing parameters are summarized in Supplementary Table M1.

## 5. Evaluation details not expanded in the main text

Time-dependent area under the curve (AUC) values were calculated at prespecified horizons using inverse probability of censoring weighting(Heagerty et al., 2000). The 36-month time point was used for the main receiver operating characteristic comparisons. For fixed-horizon analyses, patients censored before 36 months without a PFS event were excluded because their 36-month event status could not be determined. AUC gains were calculated as within-cohort differences between models sharing the same endpoint definition, and DeLong tests were used for paired AUC comparisons where applicable(DeLong et al., 1988).

Calibration curves compared predicted and observed 36-month event probabilities. Decision-curve analysis was used to estimate net benefit across threshold probabilities(Vickers and Elkin, 2006). Independent prognostic value was evaluated using Cox proportional hazards models. Because the PRISM risk score could have a nonlinear association with PFS, it was modeled using restricted cubic spline terms with 3 degrees of freedom. The proportional hazards assumption was assessed using Schoenfeld residuals(Grambsch and Therneau, 1994). The computational environment and main software packages used for model training, evaluation, and visualization are summarized in Supplementary Table M2.

## 6. Prototype-resolved interpretation outputs

Pathway-prototype association analysis quantified cross-modal relationships between pathway programs and learned morphologic prototypes. For heatmap visualization, pathway-prototype scores were standardized within pathway rows:

$$z_{p,k}=\frac{s_{p,k}-\mu_{p}}{\sigma_{p}},$$

(S6)

where $s_{p,k}$ denotes the pathway-prototype score for pathway $p$ and prototype $k$, and $\mu_{p}$ and $\sigma_{p}$ denote the mean and standard deviation of scores across prototypes for pathway $p$. This row-wise standardization was used only for visualization.

For pathway prioritization, cohort-specific pathway-prototype alignment support was combined as:

$$S_{p}^{\mathrm{align}}=0.55S_{p}^{\mathrm{TCGA}}+0.45S_{p}^{\mathrm{CPTAC}},$$

(S7)

where $S_{p}^{\mathrm{TCGA}}$ and $S_{p}^{\mathrm{CPTAC}}$ denote cohort-specific pathway-prototype softmax alignment scores. Pearson correlation was used separately to summarize cross-cohort concordance of alignment scores and was not itself the definition of the alignment score.

Representative prototype-associated patches were reviewed to assign morphologic interpretations to the learned prototypes. P1, P2, and P3 were retained as primary interpretable prototypes based on recurrent histomorphologic patterns and pathway associations. P4-associated patches were predominantly associated with fragmented peripheral tissue, tissue-edge regions, low-content tissue, and artifact/debris-associated patches; therefore, P4 was treated as a low-content/artifact-associated prototype and was not used as a primary biological interpretation anchor.

Patient-level prototype burden was defined using the locked Fig. 5F representative-patch burden workflow. For patient $i$ and prototype $k$, patches were ranked by assignment score $a_{i,j,k}$, and the top 10 representative patches were selected. Prototype burden was calculated as:

$$B_{i,k}=\frac{1}{10}\sum_{j\in Top10\left( i,k \right)} a_{i,j,k},$$

(S8)

where Top10$\left( i,k \right)$ denotes the 10 representative patches with the highest assignment scores to prototype $k$ for patient $i$. Prototype burden was compared between PRISM-defined high-risk and low-risk groups. External prototype-burden results in CPTAC-UCEC were interpreted primarily for directional consistency because of the smaller external cohort and fewer PFS events.

## 7. Risk-associated pathway-score analysis

Risk-associated pathway programs were evaluated using cohort-appropriate pathway-level quantities. In TCGA-UCEC, pathway differences were calculated from RNA-seq-derived pathway activity values. In CPTAC-UCEC, external directional comparison used PRISM-derived pathway-score values rather than independently estimated bulk RNA-seq pathway activity. For a pathway $p$, the standardized high-versus-low difference was calculated as:

$$d_{p}=\frac{\bar{x}_{p,high}-\bar{x}_{p,low}}{s_{p,pooled}},$$

(S9)

where

$$s_{p,pooled}=\sqrt{\frac{\left( n_{\mathrm{high}}-1 \right)s_{p,high}^{2}+\left( n_{\mathrm{low}}-1 \right)s_{p,low}^{2}}{n_{\mathrm{high}}+n_{\mathrm{low}}-2}}.$$

(S10)

For TCGA-UCEC, $x_{p}$ denotes RNA-seq-derived pathway activity. For CPTAC-UCEC, $x_{p}$ denotes PRISM-derived pathway-score values. These CPTAC pathway-score differences were used to assess directional concordance and cross-cohort pathway-score consistency, not as direct bulk-transcriptomic pathway activity estimates. Cross-cohort concordance between TCGA-UCEC pathway activity differences and CPTAC-UCEC PRISM-derived pathway-score differences was assessed using Spearman correlation across the evaluated pathway programs.

## 8. Prototype-number sensitivity analysis

Prototype-number sensitivity analyses compared the interpretability and held-out discrimination of $K=4$, $K=8$, and $K=16$ prototype settings. These analyses assessed pathway-prototype allocation patterns, pathway-anchor concentration, risk-associated prototype-burden patterns, and held-out discrimination in the TCGA-UCEC internal validation and CPTAC-UCEC external validation cohorts. The $K=4$ setting was selected as the primary model because it provided a compact prototype-pathway anchor structure, interpretable burden patterns, and held-out discrimination comparable to larger prototype sets.

# References

Cancer Genome Atlas Research, N., Kandoth, C., Schultz, N., Cherniack, A.D., Akbani, R., Liu, Y., et al. (2013). Integrated genomic characterization of endometrial carcinoma. *Nature* 497**,** 67–73.10.1038/nature12113

Cox, D.R. (1972). Regression Models and Life-Tables. *Journal of the Royal Statistical Society. Series B (Methodological)* 34**,** 187–220

Delong, E.R., Delong, D.M., and Clarke-Pearson, D.L. (1988). Comparing the areas under two or more correlated receiver operating characteristic curves: a nonparametric approach. *Biometrics* 44**,** 837–845

Dou, Y., Kawaler, E.A., Cui Zhou, D., Gritsenko, M.A., Huang, C., Blumenberg, L., et al. (2020). Proteogenomic Characterization of Endometrial Carcinoma. *Cell* 180**,** 729–748 e726.10.1016/j.cell.2020.01.026

Grambsch, P.M., and Therneau, T.M. (1994). Proportional hazards tests and diagnostics based on weighted residuals. *Biometrika* 81**,** 515–526.10.1093/biomet/81.3.515

Heagerty, P.J., Lumley, T., and Pepe, M.S. (2000). Time-dependent ROC curves for censored survival data and a diagnostic marker. *Biometrics* 56**,** 337–344.10.1111/j.0006-341x.2000.00337.x

Liberzon, A., Birger, C., Thorvaldsdottir, H., Ghandi, M., Mesirov, J.P., and Tamayo, P. (2015). The Molecular Signatures Database (MSigDB) hallmark gene set collection. *Cell Syst* 1**,** 417–425.10.1016/j.cels.2015.12.004

Ogata, H., Goto, S., Sato, K., Fujibuchi, W., Bono, H., and Kanehisa, M. (1999). KEGG: Kyoto Encyclopedia of Genes and Genomes. *Nucleic Acids Res* 27**,** 29–34.10.1093/nar/27.1.29

Subramanian, A., Tamayo, P., Mootha, V.K., Mukherjee, S., Ebert, B.L., Gillette, M.A., et al. (2005). Gene set enrichment analysis: a knowledge-based approach for interpreting genome-wide expression profiles. *Proc Natl Acad Sci U S A* 102**,** 15545–15550.10.1073/pnas.0506580102

Vickers, A.J., and Elkin, E.B. (2006). Decision curve analysis: a novel method for evaluating prediction models. *Med Decis Making* 26**,** 565–574.10.1177/0272989X06295361
